# Anatomical Variations of the Gallbladder and Bile Ducts: An MRI Study

**DOI:** 10.1155/2024/3877814

**Published:** 2024-10-19

**Authors:** Kigundu Yason, Kirum Gonzaga Gonza, Okello Michael, Buwembo William, Ian Munabi, Kawooya Michael

**Affiliations:** ^1^Department of Anatomy, School of Biomedical Sciences, Makerere University College of Health Sciences, Kampala, Uganda; ^2^Department of Radiology, Mengo Hospital, Kampala, Uganda

## Abstract

**Background:** The anatomy of the biliary tree is complex with anatomical variations which can be found in ~50% of the patients.

**Purpose:** Existing research on these variations primarily stems from studies in developed countries, with uncertain applicability to the Ugandan population due to noted ethnic differences in incidence rates.

**Objective:** The study was aimed at describing the anatomical variations of the gallbladder and bile ducts.

**Methods:** This retrospective cross-sectional study conducted at Kampala MRI Centre from January 2017 to December 2022 analyzed MRCP images and reports from 231 patients to document gallbladder and bile duct variations.

**Results:** 53.2% of patients exhibited typical cystic duct anatomy, and 51% had Type 1 variations in intrahepatic ducts. Importantly, the study identified a significant correlation between age and common bile duct diameter.

**Conclusion:** The findings showed various anatomical variations that were slightly higher in the study population than those reported in the literature. This study emphasizes the critical need for comprehensive knowledge to enhance surgical safety, minimize iatrogenic trauma, and improve the accuracy of diagnostic imaging and hepatobiliary procedures.

## 1. Introduction

Anatomical variations of the intrahepatic and extrahepatic bile ducts can be seen in about 50% of people [1]. Aside from predisposing to bile duct stones, recurrent pancreatitis, cholangitis, and biliary malignancies, these variations also increase the risk of bile duct iatrogenic trauma during cholecystectomy [2]. It is essential to have an accurate understanding of the anatomy and all possible variations of the biliary tree since it is critical to the success and safety of laparoscopic cholecystectomy, orthotopic liver transplantation, segmentectomy for hepatic tumors, or any other surgery on the hepatobiliary system [3].

During laparoscopy, limited knowledge of these variations is responsible for catastrophic bile duct injuries. Radiologists and other imaging professionals need a solid understanding of biliary anatomy to correctly interpret diagnostic images produced by new and older imaging technologies.

Currently, the most commonly available and used noninvasive modalities in examining the biliary system include ultrasound (US), computed tomography (CT), and magnetic resonance imaging (MRI). The most accurate and noninvasive imaging technique for demonstrating bile duct anatomy is magnetic resonance pancreatography (MRCP), which allows imaging in multiple planes (axial, sagittal, and coronal). This allows for a comprehensive assessment of the complex three-dimensional anatomy of the biliary system [4].

The main purpose of this study was to demonstrate the various anatomical variants of the gallbladder, extrahepatic, and intrahepatic ducts using magnetic resonance cholangiopancreatography and their corresponding frequencies in the Ugandan population since no previous research has ever been carried out regarding the same.

## 2. Materials and Methods

### 2.1. Study Design

This was a cross-sectional study carried out using quantitative data collection methods.

### 2.2. Study Setting

The study was conducted at Kampala MRI Centre (KAMRIC), located in Mengo along Balintuma Road. KAMRIC is a private facility that provides 1.5 Tesla MRI services in Uganda, using 1.5 Tesla Achieva MRI equipment. It offers one of the best MRI imaging quality services in Uganda. Imaging procedures are performed by experienced radiologists and imaging technologists. At this study site, MRCP procedures started in January 2017; between then and December 2022, there were a total of 420 patient images in the archive. Patient MRCP results and reports are stored on secure electronic systems which are only accessed by the radiologist or the record officer.

### 2.3. Study Population

This study involved a review of archived MRCP images of patients investigated from January 2017 to December 2022 with no age restriction.

### 2.4. Eligibility Criteria

All MRCP images of patients investigated using the MRCP technique for various indications were considered in the study.

MRCP images of patients who have undergone any surgery of the biliary tree were excluded from the study. Images missing a full representation of the biliary system were also excluded.

### 2.5. Data Collection

Under the guidance of a radiologist and imaging technologist, I reviewed, read, and interpreted the anatomical variations on the patient's MRCP images. The images used were coronal 3D images because they give a better visualization of the biliary system. To measure the common bile duct (CBD) diameter, caliper measurements were used, and they involved using digital measurement tools available in image viewing software to manually measure the diameter. The CBD was identified on the MRCP image, and calipers were used to measure the distance between the walls of the duct at its midpoint. Reports were also reviewed to identify age, gender, clinical features like jaundice, and history of surgery.

A data extraction tool was used to capture the variations and all required parameters. This data collection tool was in the form of an Excel spreadsheet comprised of organized data sets, categorized by the year of MRCP procedure, consisting of parameters like patient ID number, age, gender, gallbladder variation, cystic duct variation, intrahepatic duct variation, CBD diameter, existing biliary pathologies, and indications.

Quality control was achieved by implementing a double data entry process and periodical cross-verification of data entries with the source documents (MRCP images and reports) and implementing a review and approval process where a radiologist and imaging technologist validates the accuracy of the data set before it is used for analysis.

### 2.6. Sample Size Determination

A sample size calculation formula by Kish Leslie was used, where *N* is the sample size estimate, *P* is the assumed prevalence of anatomical variations which can be estimated to be 50.0% due to lack of availability of such parameters from a known study in the Ugandan setting, 1‐*P* is the probability of not having anatomical variation, so 1‐*P* = 50.0%, *Zα* is the standard normal deviate at 95% confidence interval corresponding to 1.96, and *δ* is the error between the estimated and true population prevalence of anatomical variations 5%.

The calculated sample size was 384.16 samples~384 samples.

Based on the exclusion criteria, the sample size was slightly reduced to 231.

### 2.7. Ethical Consideration

Ethical approval was granted by Makerere University School of Biomedical Sciences Higher Degrees Research and Ethics Committee (approval number: SBS-2022-185). A waiver of consent was also obtained from the Makerere University School of Biomedical Sciences Higher Degrees Research and Ethics Committee. Administrative clearance was granted from the management of KAMRIC. Patient data was deidentified before the study.

### 2.8. Data Management and Analysis

#### 2.8.1. Data Management

Using the extraction tool, data was entered and cleaned using Microsoft Excel. Double entry was done to ensure consistency.

#### 2.8.2. Data Analysis

Objective 1: Frequency and proportions were used to describe the variations of the gallbladder. Objective 2: Frequencies and proportions were used to describe the variations of the intrahepatic and extrahepatic ducts.

Objective 3: A correlation analysis using STATA Version 15 was done to determine the relationship between age and CBD measurements. A Pearson's coefficient was used to quantify the strength of the relationship.

## 3. Results

### 3.1. Demographic Characteristics

A total of 231 patient records (MRI images and reports) were reviewed as summarized in the patient flow diagram above. The majority (57%) of patients had a mean age of 47 years. The mean CBD diameter was 4.7 mm, and a greater majority (96%) had a normal CBD diameter, as shown in Table 1. Supporting information has been attached to show the complete data sets obtained during the data collection process.

### 3.2. Gallbladder Variations

**Figure 1:**
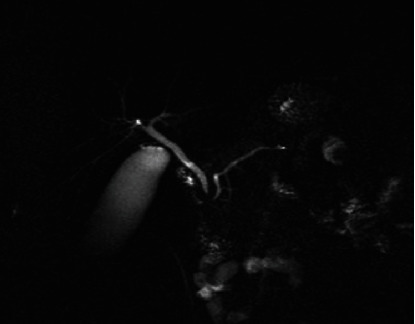
Coronal 3D MRCP image showing a cylindrical-shaped gallbladder.

**Figure 2:**
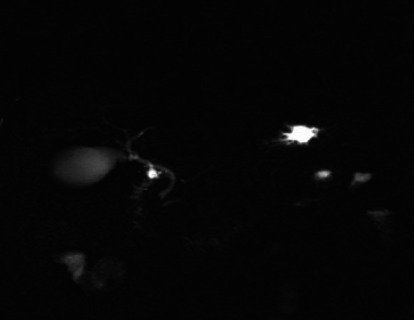
Coronal 3D MRCP image showing a pear-shaped gallbladder.

**Figure 3:**
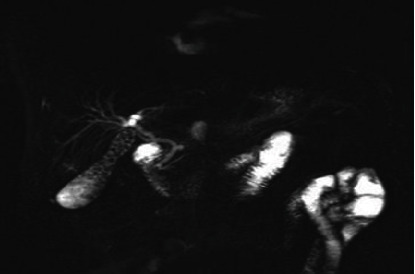
Coronal 3D MRCP image showing an hourglass-shaped gallbladder.

Most images (56.3%) had pear-shaped gallbladders, as shown in Table 2. Some of the various gallbladder shapes are shown in Figures 1, 2, and 3.

**Figure 4:**
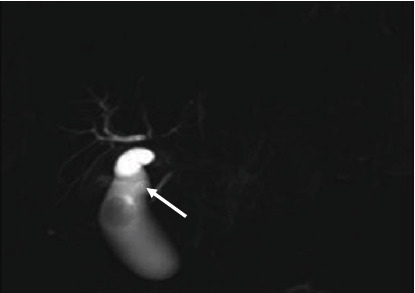
Coronal 3D MRCP image showing a gallbladder with a Hartmann's pouch (arrow).

**Figure 5:**
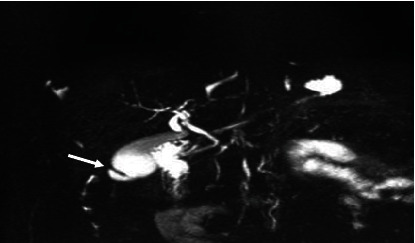
Coronal 3D MRCP image showing a gallbladder with a Phrygian cap (arrow).

Regarding the external variations of the gallbladder, 16.5% of the images had Phrygian cap gallbladders, whereas 11.3% had gallbladder with Hartmann's pouch. Most patients (72.2%) had no external variation of the gallbladder, as shown in Table 3. Figures 4 and 5 show some of the different external variations of the gallbladder observed in this study.

Regarding gallbladder position, no variation was reported because all patient MRCP images revealed gallbladders in the typical anatomical position (subhepatic position).

### 3.3. Extrahepatic Bile Duct (Cystic Duct) Variations

**Figure 6:**
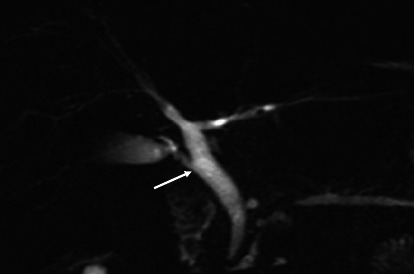
Coronal 3D MRCP image demonstrating the typical insertion/entry of cystic duct as it joins the common hepatic duct about halfway between the porta hepatis and the ampulla of Vater (arrow).

**Figure 7:**
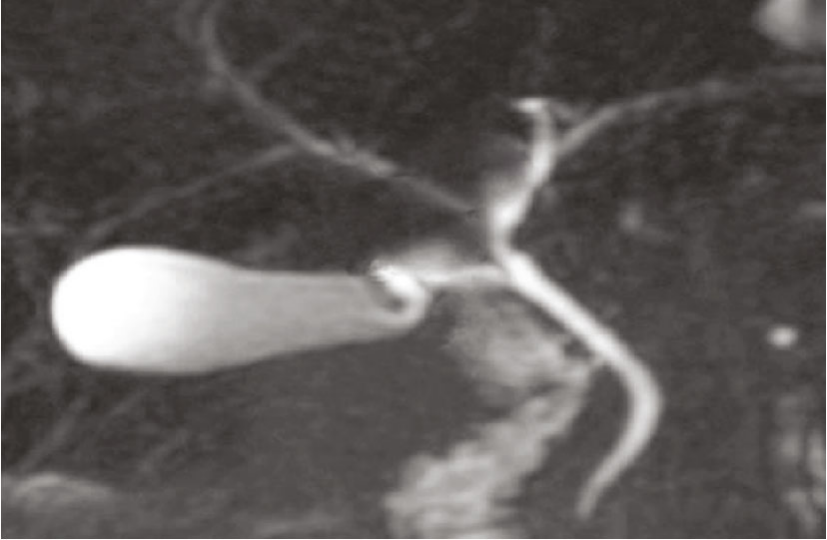
Coronal 3D MRCP image demonstrating a “high insertion” of cystic duct (the cystic duct joins the common hepatic duct close to porta hepatis).

**Figure 8:**
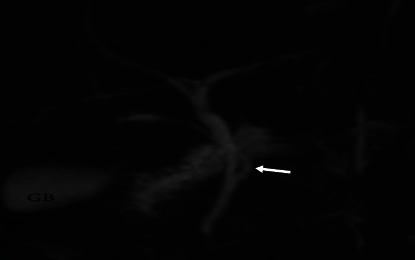
Coronal 3D MRCP image demonstrating a spiral course of the cystic duct with medial insertion. GB stands for gallbladder.

**Figure 9:**
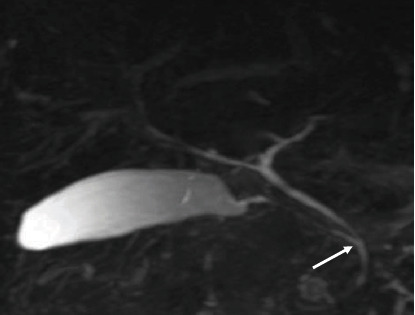
Coronal 3D MRCP image demonstrating a low insertion cystic duct (the cystic duct joins close to the duodenal papilla [arrow]).

The majority (53.2%) of the images had the “typical” extrahepatic bile duct variation. The other variations are shown in Table 4 and in Figures 6, 7, 8, and 9.

### 3.4. Intrahepatic Bile Duct Variations

**Figure 10:**
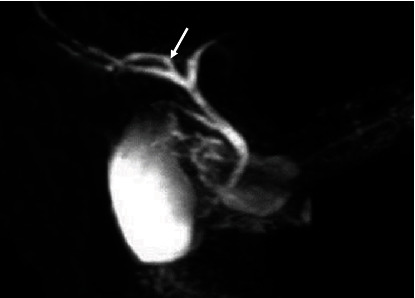
Coronal 3D MRCP image demonstrating a Type A1 variation where the right anterior hepatic duct (arrow) and right posterior hepatic duct join together to form the right hepatic duct.

**Figure 11:**
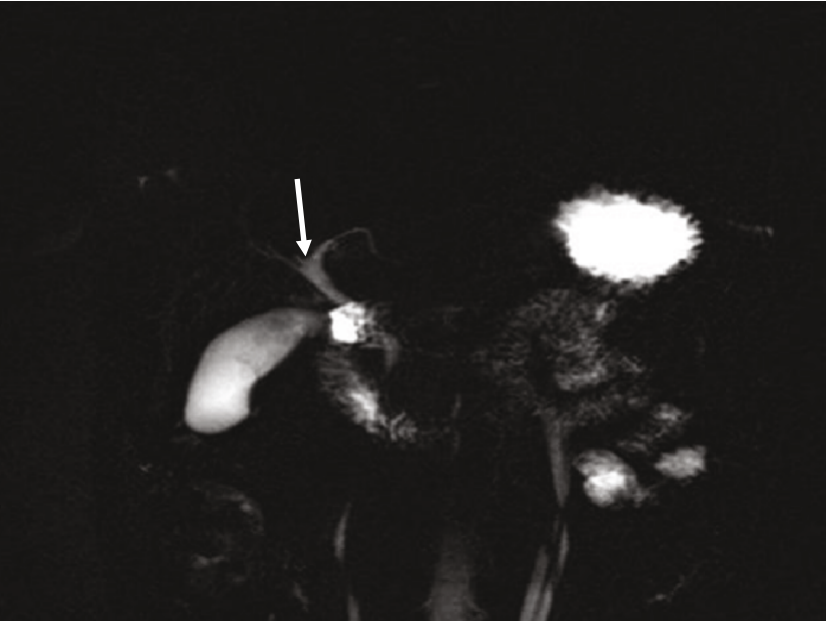
Coronal 3D MRCP image demonstrating a Type A2 variation where the right anterior hepatic duct is absent and joins directly to the confluence with the left hepatic duct to form the common hepatic duct; triple confluence (arrow).

**Figure 12:**
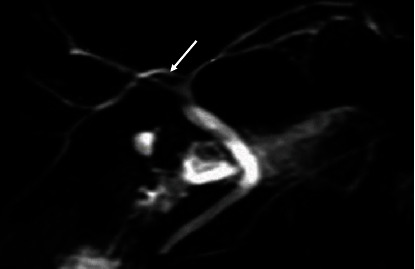
Coronal 3D MRCP image demonstrating a Type A3 variation where the right posterior hepatic duct (arrow) opens directly into the left hepatic duct to form the common hepatic duct.

**Figure 13:**
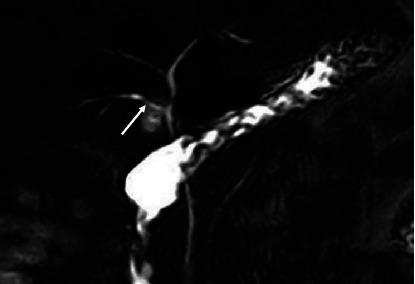
Coronal 3D MRCP image demonstrating a Type A5 variation where the right posterior hepatic duct (arrow) opens directly into the common hepatic duct.

**Figure 14:**
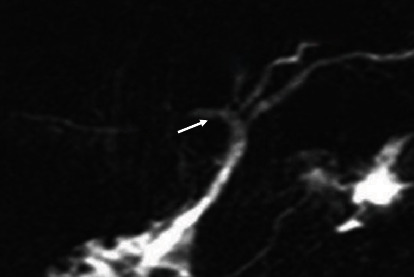
Coronal 3D MRCP image demonstrating a rare variant where Segment 2, Segment 3, and Segment 4 ducts join the right hepatic duct (arrow); there is no left hepatic duct.

All intrahepatic duct variations in this current study were classified according to the Huang et al. classification pattern [5]. Most images (118, 51%) had a Type A1 variation, while only one image (0.4%) had a very uncommon type of variation, as shown in Table 5. MRCP images showing intrahepatic variations are shown in Figures 10, 11, 12, 13, and 14.

### 3.5. Relationship Between Age and CBD Dimensions

The mean CBD diameter was 4.7 mm (standard deviation: 1.4; range: 1.6–8.5).

Almost all images (96%) had a normal diameter, as shown in Table 6.

**Figure 15:**
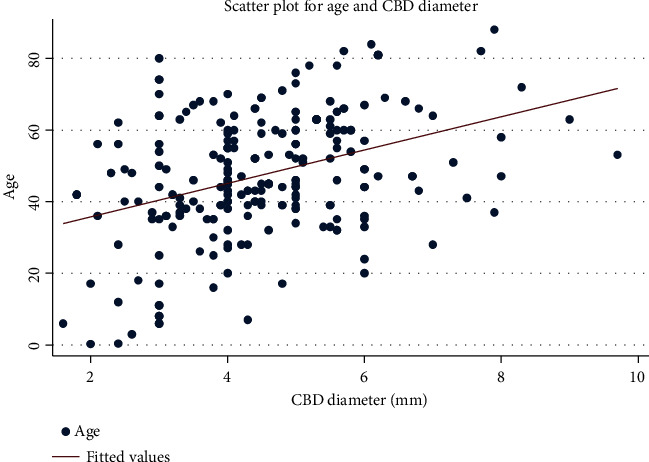
Scatter plot showing the linear relationship between age and common bile duct (CBD) diameter.

There was a positive linear relationship between age and CBD diameter, as shown in the scatter plot (Figure 15). From the scatter plot, it was noted that for every unit increase in the average age, there is a significant increase in the average CBD diameter (*R* = 0.3775, *p* < 0.0001).

## 4. Discussion

### 4.1. Gallbladder Variations

The results of this study revealed a variety of anatomical variants relating to gallbladder shape and external appearances. 130/231 (56.3%) of the MRCP images had pear-shaped gallbladders, which is a slightly lower frequency than the findings of Nadeem [6], who obtained pear-shaped gallbladders in 58/70 (82%) of the cadavers used in his study at Ajman University in western Asia. This study further demonstrates a folded fundus (Phrygian cap), which was noted in 38/231 (16.5%) of the MRCP images. The incidence was substantially lower in 3/60 (5%) of the patients in Roy et al.'s [7] cadaveric study conducted at Krishna Institute of Medical Sciences, India, which dramatically contrasts with the findings of this study.

Furthermore, this study also demonstrated gallbladders with Hartmann's pouch in only 26/231 (11.3%) of the MRCP images. This low frequency is in agreement with the findings of many other researchers including Nadeem [6], whose cadaveric study carried out at Ajman University, western Asia, reported 5/70 (7.1%) of gallbladders with Hartmann's pouch.

Results from this study indicate a relatively low incidence of a “low insertion cystic duct pattern” in only 42/231 of the cases (18.2%) which is in agreement with another MRCP study carried out in İstanbul Medeniyet University by Gündüz and Atalay [8] which revealed a “low insertion” cystic duct variation in 18/183 of the patients (9.8%).

This study also revealed a significantly high incidence of “high insertion cystic duct pattern” in 56/231 (24.2%) of the cases as compared to findings published by other scholars like Sarawagi and Shyam [9] whose MRCP study carried out at Mahatma Gandhi Medical College and Research Institute, India, showed 11/198 (5.5%) of the cases with “high insertion” cystic duct pattern.

Additionally, this study shows a significantly low incidence of a “medial insertion cystic duct pattern” in only 10/231 (4.4%) of the cases. This low frequency is compared to studies published by other scholars like Gündüz and Atalay [8], whose MRCP study was carried out at İstanbul Medeniyet University, Turkey, where they reported a slightly higher frequency of “medial insertion” cystic ducts in 38/138 (19.1%) of the cases. Furthermore, another MRCP study carried out by Sarawagi and Shyam [9] at Mahatma Gandhi Medical College and Research Institute, India, also showed a slightly higher frequency of this variant in 32/198 (16.1%) of the cases. All these studies differed in sample size and demographic characteristics, which might contribute to the variations in findings.

All gallbladder variations arise as a result of a deviation in normal anatomical development. Normally, during embryological development, the Forkhead Box f1 gene (foxf) must typically function to allow gallbladder development and maturation. Therefore, gallbladder deformity is caused by haploinsufficiency of Foxf1, which could account for the fact that some people may have gallbladder variants [10]. Gallbladders with Phrygian caps are thought to have developed as a result of localized gallbladder wall thickening. Hartmann's pouches, which resemble sacculations in the gallbladder neck and are typically caused by adhesions between the cystic duct and the gallbladder neck, are another common condition in gallbladders. A sizable Hartmann's pouch may conceal the Calot's triangle and the cystic duct, which could be problematic during cholecystectomy. Gallstones may also readily form there [11].

### 4.2. Extrahepatic Duct Variations

Results from this study indicate a relatively low incidence of a “low insertion cystic duct pattern” in only 42/231 of the cases (18.2%) which is in agreement with another MRCP study carried out in İstanbul Medeniyet University by Gündüz and Atalay [8] which revealed a “low insertion” cystic duct variation in 18/183 of the patients (9.8%).

This study also revealed a significantly high incidence of “high insertion cystic duct pattern” in 56/231 (24.2%) of the cases as compared to findings published by other scholars like Sarawagi and Shyam [9], whose MRCP study was carried out at Mahatma Gandhi Medical College and Research Institute, India, showing 11/198 (5.5%) of the cases with “high insertion” cystic duct pattern.

Additionally, this study shows a significantly low incidence of a “medial insertion cystic duct pattern” in only 10/231 (4.4%) of the cases. This low frequency is compared to studies published by other scholars like Gündüz and Atalay [8], whose MRCP study was carried out at İstanbul Medeniyet University, Turkey, where they reported a slightly higher frequency of “medial insertion” cystic ducts in 38/138 (19.1%) of the cases. Furthermore, another MRCP study carried out by Sarawagi and Shyam [9] at Mahatma Gandhi Medical College and Research Institute, India, also showed a slightly higher frequency of this variant in 32/198 (16.1%) of the cases. All these studies differed in sample size and demographic characteristics, which might contribute to the variations in findings.

This study also explored the extrahepatic bile duct system, specifically the cystic duct, because a wide range of variability is mostly noticed in the cystic duct course and its junction with the extrahepatic bile duct [12]. Results from this study reveal a “low insertion cystic duct pattern” in 42/231 of the cases (18.2%). This pattern occurs due to the premature separation of pars hepatica from pars cystica, and as a result, the junction between the two future ducts (cystic duct and CBD) gets close to the duodenal papilla. It is associated with a high rate of CBD stone formation [13].

This study also revealed a significantly high incidence of “high insertion cystic duct pattern” in 56/231 (24.2%) of the cases. This may arise in case of delayed separation of the pars hepatica from pars cystica, and as a result, the junction of the cystic with the common hepatic duct will be close to the porta hepatis, or at the confluence of the left and right hepatic ducts [14]. High-entry cystic ducts may increase the risk of injury of either the right or left hepatic duct during cystic duct ligation. Additionally, this study shows a significantly low incidence of “medial insertion cystic duct pattern” in only 10/231 (4.4%) of the cases. This type of variation occurs due to embryological malrotation of the cystic duct as a result of the faulty transfer of the choledochoduodenal junction during the rotation of the duodenum [15]. The twist of the duct during its formation may be either clockwise or counterclockwise, causing the cystic duct to take a spiral course either anterior or posterior to the common hepatic duct [16].

### 4.3. Intrahepatic Duct Variations

This study discovered that the typical pattern (Type A1) was observed in 118/231 (51%) of the cases, and it has been described as the most prevalent variant by many other scholars, including Tawab and Ali [17] in an MRCP study conducted at the National Hepatology Research Institute, Cairo, Egypt, where he reported 207/353 (58.6%) of the cases. Furthermore, atypical branching patterns of intrahepatic bile ducts were revealed. These include Type A2 or triple confluence patterns where the RASD, RPSD, and LHD join simultaneously to form the CHD in 69/231 (29.9%) of the cases. This trifurcation pattern was also reported by Sarawagi and Shyam [9] in an MRCP study with a significantly lower frequency of 26/224 (11.6%) than in this current study. Additionally, this study also reveals Type A3 variation in 34/231 (14.7%) of the cases which is almost similar to an MRCP study by Khanduja, Chawla, and Diwan [18] carried out at Indira Gandhi Medical College Shimla, India, where findings showed 7/56 (12.5%) of the cases of Type A3 variation. Findings from this study also noticed the Type A5 variant which involves ectopic drainage of RPSD into the common hepatic duct in only 9/231 (4%) of the cases. This is in agreement with Mandooh, Abdelbary, and El's MRCP-based study carried out at the National Hepatology Research Institute, Cairo, where he also found this variant in only 14/353 of the cases (4%) (Tawab and Ali [17]). This study also denotes a solitary finding of a very uncommon variant which involves ectopic drainage of segmental ducts (II, III, and IV) directly into the RHD in only 1/231 of the cases (0.4%).

According to Krupczak-Hollis et al. [19], intrahepatic variations have a genetic basis. In their genetic study, they reported that the Forkhead Box m1 transcription factor (Foxm1b) is necessary for normal intrahepatic bile duct cell development from the ductal plate in addition to regulating the development of vessels. Thus, a defect in the ductal plate's remodeling leads to haploinsufficiency and can result in irregularities in the intrahepatic ducts' branching pattern.

### 4.4. CBD Diameter and Age

In this present study, it was found that there is a statistically significant relationship between age and CBD diameter; for every unit increase in the average age, there is an increase in the average CBD diameter. Several published literatures exist about the normal size of the CBD, but only a few reports consider the important age-dependent variations in CBD diameter. To diagnose obstructive biliary disease, recognition of bile duct dilatation is required. The normal caliber of the CBDs is expected to be less than 6.0 mm sonographically and 8.0 mm cholangiographically [20]. According to this present study, there is a physiological increase of 0.5–1 mm per decade, as also suggested by many other scholars [21, 22], with an upper normal limit of 8.5 mm in the elderly. Previous studies have shown that the mean diameter of the CBD is between 3.4 and 7.39 mm, with a range of 1.0–15.0 mm [23], and results in this study were well within the reported range. CBD diameters are significantly different between patients who are younger or older than 60 years of age, perhaps because of the fragmentation of the smooth muscle bands accompanied by the loss of the reticuloendothelial network of the CBD ductal wall over time [24].

The increase in CBD diameter with age occurs as a result of the characteristic fragmentation of the longitudinal smooth myocyte bands and interspersed connective tissue combined with the decrease in the reticular elastic framework of the duct wall over time which leads to reduced contractility and hypotonus of the CBD [24]. Additionally, drugs such as calcium antagonists and nitroglycerine, which are frequently taken by the elderly population, may influence the contractility and tonus of the duct wall [25].

The differences in the frequencies of biliary anatomical variations between this study and others can be attributed to population-specific genetic factors. According to Downing et al. [26], variability in biliary anatomy is often linked to ethnic differences.

## 5. Conclusion

Biliary anatomical variations are prevalent at KAMRIC. Based on our research findings, key recommendations have been identified to enhance radiological and surgical practices in managing hepatobiliary pathologies. Thorough preoperative imaging and planning should be carried out to address common hepatobiliary variants such as Phrygian cap gallbladders, Hartmann's pouch gallbladders, cystic duct variants, and intrahepatic duct variations to reduce the risk of surgical errors.

Additionally, it is essential to provide comprehensive training for radiologists and surgeons on hepatobiliary variations to improve diagnostic accuracy and surgical outcomes. Implementing standardized protocols for reporting anatomical variations in MRCP images is crucial for enhancing consistency and accuracy in preoperative assessments. It is also vital to consider age-related variations in common bile duct diameter during clinical evaluation of patients suspected of choledocholithiasis.

The utilization of advanced imaging techniques for detailed 3D reconstructions is crucial in supporting surgical planning and identifying anatomical variants. Lastly, fostering multidisciplinary collaboration among radiologists, hepatobiliary surgeons, and gastroenterologists through regular case discussions is essential for optimizing patient care.

### 5.1. Study Limitations

Data was from one health facility; therefore, it may not be generalized to the whole population.

A nonrandomized sample was used due to limited images in the study site. This may introduce selection bias in the study, leading to over- or underreporting of biliary duct variation.

## Figures and Tables

**Table 1:** Demographic characteristics of patients at Kampala MRI Centre.

**Variable**	**Frequency**	**Percentage**
Sex		
Male	99	43
Female	132	57
Age		
0.2–18	17	7.4
19–30	13	5.6
31–40	48	20.8
41–50	53	22.9
50 and above	100	43.3
CBD diameter		
1–7 mm	221	96
7.1 mm and above	10	4

**Table 2:** Variations in gallbladder shape among patients at Kampala MRI Centre.

**Gallbladder shape**	**Frequency**	**Percentage**
Cylindrical	76	32.9
Hourglass shaped	25	10.8
Pear shaped	130	56.3
Total	231	100

**Table 3:** External variations of the gallbladder among patients at Kampala MRI Centre.

**External variation**	**Frequency**	**Percentage**
Hartmann's pouch	26	11.3
Phrygian cap	38	16.5
Gallbladders without external variations	167	72.2
Total	231	100

**Table 4:** Extrahepatic bile duct variations among patients at Kampala MRI Centre.

**Extrahepatic bile duct variations**	**Frequency**	**Percentage**
High	56	24.2
Low	42	18.2
Medial	10	4.4
Normal	123	53.2
Total	231	100

**Table 5:** Intrahepatic bile duct variations among patients at Kampala MRI Centre.

**Intrahepatic bile duct variations**	**Frequency**	**Percentage**
Type 1	118	51
Type 2	69	29.9
Type 3	34	14.7
Type 4	9	4
Others	1	0.4
Total	231	100

**Table 6:** Common bile duct measurements among patients at Kampala MRI Centre.

**Variable (CBD diameter)**	**Frequency**	**Percentage**
1–7 mm	221	96
7.1 mm and above	10	4

## Data Availability

Patient MRCP images and reports are stored on secure electronic systems only accessed by the radiologists and record officers of Kampala MRI Centre. PDF files have been attached to show the data collected from the Kampala MRI Centre archives (MRCP images and reports).

## Supporting Information

Various files have been attached to the system to show the data collected from the archives of Kampala MRI Centre. These include the following:

Supporting Information 1 2017 data set PDF file which contains data collected from MRCP images and reports of patients who visited Kampala MRI Centre in the year 2017.

Supporting Information 2 2018 data set PDF file which contains data collected from MRCP images and reports of patients who visited Kampala MRI Centre in the year 2018.

Supporting Information 3 2019 data set PDF file which contains data collected from MRCP images and reports of patients who visited Kampala MRI Centre in the year 2019.

Supporting Information 4 2020 data set PDF file which contains data collected from MRCP images and reports of patients who visited Kampala MRI Centre in the year 2020.

Supporting Information 5 2021 data set PDF file which contains data collected from MRCP images and reports of patients who visited Kampala MRI Centre in the year 2021.

Supporting Information 6 2022 data set PDF file which contains data collected from MRCP images and reports of patients who visited Kampala MRI Centre in the year 2022.
