# Supplementary material for: Anatomical Variations of the Gallbladder and Bile Ducts: An MRI Study
Source: Int J Hepatol. 2024 Oct 19;2024:3877814. doi: 10.1155/2024/3877814 (PMC11512644; doi:10.1155/2024/3877814)
Supplement: Supporting Information 1 — 2017 data set PDF file which contains data collected from MRCP images and reports of patients who visited Kampala MRI Centre in the year 2017. [file 3877814.f1.pdf]

2017 DATA SET

Patient ID	Age
2	46,F
6	17,M
8	78,M
10	49,M
12	46,M
13	57, M
15	53,F
16	33,F
18	69,F
19	55,F
20	33,F
21	88,M
24	58,F
25	68, M
28	6, F
31	53,F
32	71,M
33	68,F
34	53,M
35	39,M
36	51,M
37	41,M
39	53,M
41	26,F
42	39,M
45	51,M
48	28,M
49	37,M
50	14,M
51	37,F
53	51,M
58	38,F
59	79,M

64	32,F
65	55,F
66	57,M
68	79,M
69	36,M
70	54,M
71	25,M
75	2months, M
77	64,F
81	39,F
83	33,M
84	72,M
87	56,F
89	64,F
90	57,F
91	44,F
94	69,F
96	16,M
101	70,M
111	42,M
112	64,F
115	52,F
117	39,M
118	52,M
120	63/F
127	63,F

Gall bladder variation (shape, anatomical position)
Cylindrical, Normal position
Pear shaped, Normal position
Pear shaped, Normal position
Pear shaped, Normal position
Pear shaped, Normal position
Pear shaped, Normal position
Pear shaped, Normal position
Pear shaped, Normal position
Pear shaped, Normal position
Pear shaped, Normal position
Pear shaped, Normal position
Pear shaped, Normal position
Hartman's pouch , Normal position
Cylindrical, Normal position
Cylindrical, Normal position
Pear shaped, Normal position
Cylindrical, Normal position
Cylindrical, Normal position
Pear shaped, Normal position
Cylindrical, Normal position
Cylindrical, Normal position
Pear shaped, Normal position
Pear shaped, Normal position
Phyrigian cap gallbladder, Normal position
Pear shaped, Normal position
Pear shaped, Normal position
Pear shaped, Normal position
Pear shaped, Normal position
Pear shaped, Normal position
Cylindrical, Normal position
Cylindrical, Normal position
Cylindrical, Normal position
Cylindrical, Normal position

Pear shaped, Normal position
Pear shaped, Normal position
Pear shaped, Normal position
Pear shaped, Normal position
Phyrigian cap gallbladder, Normal position
Pear shaped, Normal position
Cylindrical, Normal position
Cylindrical, Normal position
Pear shaped, Normal position
Pear shaped, Normal position
Pear shaped, Normal position
Pear shaped, Normal position
Pear shaped, Normal position
Pyrigian cap,Normal position
Pear shaped, Normal position
Pyrigian cap,Normal position
Pear shaped, Normal position
Pear shaped, Normal position
Pear shaped, Normal position
Pear shaped, Normal position
Pyrigian cap,Normal position
Pyrigian cap,Normal position
Pyrigian cap,Normal position
Cylindrical, Normal position
Pyrigian cap,Normal position
Pyrigian cap,Normal position

Extrahepatic bile duct variation
Normal: cystic duct joins middle a third of the combined lengths of the CBD & CHD
High entry
Normal: cystic duct joins middle a third of the combined lengths of the CBD & CHD
Low entry
Normal: cystic duct joins middle a third of the combined lengths of the CBD & CHD
Normal: cystic duct joins middle a third of the combined lengths of the CBD & CHD
Normal: cystic duct joins middle a third of the combined lengths of the CBD & CHD
Normal: cystic duct joins middle a third of the combined lengths of the CBD & CHD
Normal: cystic duct joins middle a third of the combined lengths of the CBD & CHD
Normal: cystic duct joins middle a third of the combined lengths of the CBD & CHD
Normal: cystic duct joins middle a third of the combined lengths of the CBD & CHD
Normal: cystic duct joins middle a third of the combined lengths of the CBD & CHD
High entry
High entry
High entry
High entry
Normal: cystic duct joins middle a third of the combined lengths of the CBD & CHD
Normal: cystic duct joins middle a third of the combined lengths of the CBD & CHD
High entry
Normal: cystic duct joins middle a third of the combined lengths of the CBD & CHD
Normal: cystic duct joins middle a third of the combined lengths of the CBD & CHD
Normal: cystic duct joins middle a third of the combined lengths of the CBD & CHD
Medial entry
Normal: cystic duct joins middle a third of the combined lengths of the CBD & CHD
Normal: cystic duct joins middle a third of the combined lengths of the CBD & CHD
Normal: cystic duct joins middle a third of the combined lengths of the CBD & CHD
Low entry
Normal: cystic duct joins middle a third of the combined lengths of the CBD & CHD
Normal: cystic duct joins middle a third of the combined lengths of the CBD & CHD
Normal: cystic duct joins middle a third of the combined lengths of the CBD & CHD
Normal: cystic duct joins middle a third of the combined lengths of the CBD & CHD
Normal: cystic duct joins middle a third of the combined lengths of the CBD & CHD
Normal: cystic duct joins middle a third of the combined lengths of the CBD & CHD

Normal: cystic duct joins middle a third of the combined lengths of the CBD & CHD
Normal: cystic duct joins middle a third of the combined lengths of the CBD & CHD
Normal: cystic duct joins middle a third of the combined lengths of the CBD & CHD
Normal: cystic duct joins middle a third of the combined lengths of the CBD & CHD
High entry
High entry
Normal: cystic duct joins middle a third of the combined lengths of the CBD & CHD
High entry
High entry
Low entry
High entry
Low entry
Medial entry
High entry
High entry
Normal: cystic duct joins middle a third of the combined lengths of the CBD & CHD
Normal: cystic duct joins middle a third of the combined lengths of the CBD & CHD
High entry
Normal: cystic duct joins middle a third of the combined lengths of the CBD & CHD
Normal: cystic duct joins middle a third of the combined lengths of the CBD & CHD
High entry
Normal: cystic duct joins middle a third of the combined lengths of the CBD & CHD
Normal: cystic duct joins middle a third of the combined lengths of the CBD & CHD
Low entry
normal: cystic duct joins middle a third of the combined lengths of the CBD & CHD
High entry

Intrahepatic bile duct variation
Type 3 RPSD joins the LHD ,RASD joins the LHD to form CHD
Type 1 RASD joins the RPSD to form the RHD, RHD joins LHD to form the CHD
Type 2 (Triple confluence) RASD, RPSD and LHD join simultaneously to form the CHD
Type 1 RASD joins the RPSD to form the RHD, RHD joins LHD to form the CHD
Type 2 (Triple confluence) RASD, RPSD and LHD join simultaneously to form the CHD
Type 1 RASD joins the RPSD to form the RHD, RHD joins LHD to form the CHD
Type 3 RPSD joins the LHD ,RASD joins the LHD to form CHD,
Type 1 RASD joins the RPSD to form the RHD, RHD joins LHD to form the CHD
Type 1 RASD joins the RPSD to form the RHD, RHD joins LHD to form the CHD
Type 1 RASD joins the RPSD to form the RHD, RHD joins LHD to form the CHD
Type 2 (Triple confluence) RASD, RPSD and LHD join simultaneously to form the CHD
Type 1 RASD joins the RPSD to form the RHD, RHD joins LHD to form the CHD
Type 1 RASD joins the RPSD to form the RHD, RHD joins LHD to form the CHD
Type 2 (Triple confluence) RASD, RPSD and LHD join simultaneously to form the CHD
Type 1 RASD joins the RPSD to form the RHD, RHD joins LHD to form the CHD
Type 3 RPSD joins the LHD ,RASD joins the LHD to form CHD,
Type 1 RASD joins the RPSD to form the RHD, RHD joins LHD to form the CHD
Type 1 RASD joins the RPSD to form the RHD, RHD joins LHD to form the CHD
Type 3 RPSD joins the LHD ,RASD joins the LHD to form CHD,
Type 2 (Triple confluence) RASD, RPSD and LHD join simultaneously to form the CHD
Type 3 RPSD joins the LHD ,RASD joins the LHD to form CHD,
Type 3 RPSD joins the LHD ,RASD joins the LHD to form CHD,
Type 1 RASD joins the RPSD to form the RHD, RHD joins LHD to form the CHD
Type 3 RPSD joins the LHD ,RASD joins the LHD to form CHD,
Type 4 RPSD drains into the common hepatic duct (CHD)
Type 2 (Triple confluence) RASD, RPSD and LHD join simultaneously to form the CHD
Type 1 RASD joins the RPSD to form the RHD, RHD joins LHD to form the CHD
Type 3 RPSD joins the LHD ,RASD joins the LHD to form CHD,
Type 3 RPSD joins the LHD ,RASD joins the LHD to form CHD,
Type 2 (Triple confluence) RASD, RPSD and LHD join simultaneously to form the CHD
Type 1 RASD joins the RPSD to form the RHD, RHD joins LHD to form the CHD
Type 1 RASD joins the RPSD to form the RHD, RHD joins LHD to form the CHD
Type 2 (Triple confluence) RASD, RPSD and LHD join simultaneously to form the CHD

Type 1 RASD joins the RPSD to form the RHD, RHD joins LHD to form the CHD
Type 2 (Triple confluence) RASD, RPSD and LHD join simultaneously to form the CHD
Type 1 RASD joins the RPSD to form the RHD, RHD joins LHD to form the CHD
Type 1 RASD joins the RPSD to form the RHD, RHD joins LHD to form the CHD
Type 1 RASD joins the RPSD to form the RHD, RHD joins LHD to form the CHD
Type 1 RASD joins the RPSD to form the RHD, RHD joins LHD to form the CHD
Type 1 RASD joins the RPSD to form the RHD, RHD joins LHD to form the CHD
Type 2 (Triple confluence) RASD, RPSD and LHD join simultaneously to form the CHD
Type 3 RPSD joins the LHD ,RASD joins the LHD to form CHD,
Type 1 RASD joins the RPSD to form the RHD, RHD joins LHD to form the CHD
Type 1 RASD joins the RPSD to form the RHD, RHD joins LHD to form the CHD
Type 2 (Triple confluence) RASD, RPSD and LHD join simultaneously to form the CHD
Type 3 RPSD joins the LHD ,RASD joins the LHD to form CHD,
Type 1 RASD joins the RPSD to form the RHD, RHD joins LHD to form the CHD
Type 2 (Triple confluence) RASD, RPSD and LHD join simultaneously to form the CHD
Type 1 RASD joins the RPSD to form the RHD, RHD joins LHD to form the CHD
Type 1 RASD joins the RPSD to form the RHD, RHD joins LHD to form the CHD
Type 3 RPSD joins the LHD ,RASD joins the LHD to form CHD,
Type 1 RASD joins the RPSD to form the RHD, RHD joins LHD to form the CHD
Type 1 RASD joins the RPSD to form the RHD, RHD joins LHD to form the CHD
Type 2 (Triple confluence) RASD, RPSD and LHD join simultaneously to form the CHD
Type 2 (Triple confluence) RASD, RPSD and LHD join simultaneously to form the CHD
Type 2 (Triple confluence) RASD, RPSD and LHD join simultaneously to form the CHD
Type 1 RASD joins the RPSD to form the RHD, RHD joins LHD to form the CHD
Type 2 (Triple confluence) RASD, RPSD and LHD join simultaneously to form the CHD
Type 2 (Triple confluence) RASD, RPSD and LHD join simultaneously to form the CHD

CBD diameter midsection	Abbrevations and their meanings
5.6mm	[CBD stands for common bile duct] [CHD stands for common hepatic duct] [RASD stands for Right anterior sectoral duct] [RPSD stands for Right posterior sectoral duct] [RHD stands for right hepatic duct] [LHD stands for Left hepatic ducts] [M stands for Male] [F stands for Female]
4.8mm	
5.2mm	
3.1mm	
4.mm	
5.6mm	
9.7mm	
5.5mm	
6.3mm	
4.1mm	
5.4mm	
7.9mm	
8.0 mm	
6.6mm	
3mm	
4.6mm	
4.8mm	
5.5mm	
3.8mm	
5mm	
4mm	
3.3mm	
3.8mm	
3.6mm	
4.5mm	
dilated	
2.4mm	
2.9mm	
dilated	
7.9mm	
7.3mm	
3.4mm	
dilated	

dilated
dilated
4.1mm
dilated
3.3mm
dilated
3.8mm
2.1mm
7mm
4.3mm
3.2mm
2.4mm
3mm
4mm
5mm
4.5mm
3.8mm
3mm
1.8mm
3mm
3.9mm
3.3mm
4.4mm
5.3mm
3.3mm

2017 DATA SET

Patient ID	Age	Gall bladder variation (shape, anatomical position)	Extrahepatic bile duct variation	Intrahepatic bile duct variation	CBD diameter midsection	Abbrevations and thei
2	46,F	Cylindrical, Normal position	Normal: cystic duct joins middle a third of the combined lengths of the CBD	Type 3 RPSD joins the LHD ,RASD joins the LHD to form CHD	5.6mm	[CBD stands for commo stands for common hepa stands for Right anterior [RPSD stands for Right p duct] [RHD stands for rig [LHD stands for Left hep
6	17,M	Pear shaped, Normal position	High entry	Type 1 RASD joins the RPSD to form the RHD, RHD joins LHD to form the CH	4.8mm	
8	78,M	Pear shaped, Normal position	Normal: cystic duct joins middle a third of the combined lengths of the CBD	Type 2 (Triple confluence) RASD, RPSD and LHD join simultaneously to form t	5.2mm	
10	49,M	Pear shaped, Normal position	Low entry	Type 1 RASD joins the RPSD to form the RHD, RHD joins LHD to form the CH	3.1mm	
12	46,M	Pear shaped, Normal position	Normal: cystic duct joins middle a third of the combined lengths of the CBD	Type 2 (Triple confluence) RASD, RPSD and LHD join simultaneously to form t	4.mm	
13	57, M	Pear shaped, Normal position	Normal: cystic duct joins middle a third of the combined lengths of the CBD	Type 1 RASD joins the RPSD to form the RHD, RHD joins LHD to form the CH	5.6mm	
15	53,F	Pear shaped, Normal position	Normal: cystic duct joins middle a third of the combined lengths of the CBD	Type 3 RPSD joins the LHD ,RASD joins the LHD to form CHD,	9.7mm	
16	33,F	Pear shaped, Normal position	Normal: cystic duct joins middle a third of the combined lengths of the CBD	Type 1 RASD joins the RPSD to form the RHD, RHD joins LHD to form the CH	5.5mm	
18	69,F	Pear shaped, Normal position	Normal: cystic duct joins middle a third of the combined lengths of the CBD	Type 1 RASD joins the RPSD to form the RHD, RHD joins LHD to form the CH	6.3mm	
19	55,F	Pear shaped, Normal position	Normal: cystic duct joins middle a third of the combined lengths of the CBD	Type 1 RASD joins the RPSD to form the RHD, RHD joins LHD to form the CH	4.1mm	
20	33,F	Pear shaped, Normal position	Normal: cystic duct joins middle a third of the combined lengths of the CBD	Type 2 (Triple confluence) RASD, RPSD and LHD join simultaneously to form t	5.4mm	
21	88,M	Pear shaped, Normal position	Normal: cystic duct joins middle a third of the combined lengths of the CBD	Type 1 RASD joins the RPSD to form the RHD, RHD joins LHD to form the CH	7.9mm	
24	58,F	Hartman's pouch , Normal position	High entry	Type 1 RASD joins the RPSD to form the RHD, RHD joins LHD to form the CH	8.0 mm	
25	68, M	Cylindrical, Normal position	High entry	Type 2 (Triple confluence) RASD, RPSD and LHD join simultaneously to form t	6.6mm	
28	6, F	Cylindrical, Normal position	High entry	Type 1 RASD joins the RPSD to form the RHD, RHD joins LHD to form the CH	3mm	
31	53,F	Pear shaped, Normal position	High entry	Type 3 RPSD joins the LHD ,RASD joins the LHD to form CHD,	4.6mm	
32	71,M	Cylindrical, Normal position	Normal: cystic duct joins middle a third of the combined lengths of the CBD	Type 1 RASD joins the RPSD to form the RHD, RHD joins LHD to form the CH	4.8mm	
33	68,F	Cylindrical, Normal position	Normal: cystic duct joins middle a third of the combined lengths of the CBD	Type 1 RASD joins the RPSD to form the RHD, RHD joins LHD to form the CH	5.5mm	
34	53,M	Pear shaped, Normal position	High entry	Type 3 RPSD joins the LHD ,RASD joins the LHD to form CHD,	3.8mm	
35	39,M	Cylindrical, Normal position	Normal: cystic duct joins middle a third of the combined lengths of the CBD	Type 2 (Triple confluence) RASD, RPSD and LHD join simultaneously to form t	5mm	
36	51,M	Cylindrical, Normal position	Normal: cystic duct joins middle a third of the combined lengths of the CBD	Type 3 RPSD joins the LHD ,RASD joins the LHD to form CHD,	4mm	
37	41,M	Pear shaped, Normal position	Normal: cystic duct joins middle a third of the combined lengths of the CBD	Type 3 RPSD joins the LHD ,RASD joins the LHD to form CHD,	3.3mm	
39	53,M	Pear shaped, Normal position	Medial entry	Type 1 RASD joins the RPSD to form the RHD, RHD joins LHD to form the CH	3.8mm	
41	26,F	Phyrigian cap gallbladder, Normal position	Normal: cystic duct joins middle a third of the combined lengths of the CBD	Type 3 RPSD joins the LHD ,RASD joins the LHD to form CHD,	3.6mm	
42	39,M	Pear shaped, Normal position	Normal: cystic duct joins middle a third of the combined lengths of the CBD	Type 4 RPSD drains into the common hepatic duct (CHD)	4.5mm	
45	51,M	Pear shaped, Normal position	Normal: cystic duct joins middle a third of the combined lengths of the CBD	Type 2 (Triple confluence) RASD, RPSD and LHD join simultaneously to form t	dilated	
48	28,M	Pear shaped, Normal position	Low entry	Type 1 RASD joins the RPSD to form the RHD, RHD joins LHD to form the CH	2.4mm	
49	37,M	Pear shaped, Normal position	Normal: cystic duct joins middle a third of the combined lengths of the CBD	Type 3 RPSD joins the LHD ,RASD joins the LHD to form CHD,	2.9mm	
50	14,M	Pear shaped, Normal position	Normal: cystic duct joins middle a third of the combined lengths of the CBD	Type 3 RPSD joins the LHD ,RASD joins the LHD to form CHD,	dilated	
51	37,F	Cylindrical, Normal position	Normal: cystic duct joins middle a third of the combined lengths of the CBD	Type 2 (Triple confluence) RASD, RPSD and LHD join simultaneously to form t	7.9mm	
53	51,M	Cylindrical, Normal position	Normal: cystic duct joins middle a third of the combined lengths of the CBD	Type 1 RASD joins the RPSD to form the RHD, RHD joins LHD to form the CH	7.3mm	
[illegible]	[illegible]	[illegible]	[illegible]	[illegible]	[illegible]	
