# Supplementary material for: Anatomical Variations of the Gallbladder and Bile Ducts: An MRI Study
Source: Int J Hepatol. 2024 Oct 19;2024:3877814. doi: 10.1155/2024/3877814 (PMC11512644; doi:10.1155/2024/3877814)
Supplement: Supporting Information 2 — 2018 data set PDF file which contains data collected from MRCP images and reports of patients who visited Kampala MRI Centre in the year 2018. [file 3877814.f2.pdf]

2018 DATA SET

Patient ID	Age	Gall bladder variation (shape, position)
128	81/F	Pear shaped, Normal position
129	55/F	Cylindrical, Normal position
130	44/F	Cylindrical, Normal position
132	59/M	Cylindrical, Normal position
134	78/F	Cylindrical, Normal position
137	7/M	Cylindrical, Normal position
139	52/F	Cylindrical, Normal position
140	84/F	Cylindrical, Normal position
141	24/F	Pear shaped, Normal position
142	46/F	Pear shaped, Normal position
143	60/F	Pear shaped, Normal position
144	39/M	Phyrigian cap gallbladder, Normal position
145	6/M	Cylindrical, Normal position
148	39/F	Cylindrical, Normal position
150	45/M	Cylindrical, Normal position
154	36/M	Cylindrical, Normal position
155	52/F	Pear shaped, Normal position
156	36/M	Pear shaped, Normal position
159	48/M	Pear shaped, Normal position
160	17/F	Pear shaped, Normal position
161	34/F	Pear shaped, Normal position
162	3/M	Cylindrical, Normal position
163	63/F	Cylindrical, Normal position
164	42/M	Cylindrical, Normal position
165	52/M	Cylindrical, Normal position
166	46/F	Pear shaped, Normal position
167	42/M	Pear shaped, Normal position
168	69/F	Pear shaped, Normal position
169	38/F	Pear shaped, Normal position
170	55/F	Pear shaped, Normal position
171	81/F	Pear shaped, Normal position

Extrahepatic bile duct variation (cycstic duct)
Right lateral union of cystic duct to CHD midway between porta hepatis & ampulla of vatter
High entry
High entry
High entry
Low entry
Low entry
Medial entry (CD crosses posterior to CHD and joins it medially)
Low entry
Low entry
Low entry
Low entry
High entry
Right lateral union of cystic duct to CHD midway between porta hepatis & ampulla of vatter
Right lateral union of cystic duct to CHD midway between porta hepatis & ampulla of vatter
Right lateral union of cystic duct to CHD midway between porta hepatis & ampulla of vatter
Right lateral union of cystic duct to CHD midway between porta hepatis & ampulla of vatter
High entry
High entry
High entry
High entry
Low entry
Low entry
Low entry
Right lateral union of cystic duct to CHD midway between porta hepatis & ampulla of vatter
Right lateral union of cystic duct to CHD midway between porta hepatis & ampulla of vatter
Right lateral union of cystic duct to CHD midway between porta hepatis & ampulla of vatter
Right lateral union of cystic duct to CHD midway between porta hepatis & ampulla of vatter
Right lateral union of cystic duct to CHD midway between porta hepatis & ampulla of vatter
Right lateral union of cystic duct to CHD midway between porta hepatis & ampulla of vatter
High entry
High entry

Intrahepatic bile duct variation
Type 3 RPSD joins the LHD ,RASD joins the LHD to form CHD,
Type 1 RASD joins the RPSD to form the RHD, RHD joins LHD to form the CHD
Type 1 RASD joins the RPSD to form the RHD, RHD joins LHD to form the CHD
Type 1 RASD joins the RPSD to form the RHD, RHD joins LHD to form the CHD
Type 1 RASD joins the RPSD to form the RHD, RHD joins LHD to form the CHD
Type 1 RASD joins the RPSD to form the RHD, RHD joins LHD to form the CHD
Type 2 (Triple confluence) RASD, RPSD and LHD join simultaneously to form the CHD
Type 1 RASD joins the RPSD to form the RHD, RHD joins LHD to form the CHD
Type 4 RPSD drains into the common hepatic duct (CHD)
Type 1 RASD joins the RPSD to form the RHD, RHD joins LHD to form the CHD
Type 1 RASD joins the RPSD to form the RHD, RHD joins LHD to form the CHD
Type 2 (Triple confluence) RASD, RPSD and LHD join simultaneously to form the CHD
Type 1 RASD joins the RPSD to form the RHD, RHD joins LHD to form the CHD
Type 3 RPSD joins the LHD ,RASD joins the LHD to form CHD,
Type 3 RPSD joins the LHD ,RASD joins the LHD to form CHD,
Type 3 RPSD joins the LHD ,RASD joins the LHD to form CHD,
Type 2 (Triple confluence) RASD, RPSD and LHD join simultaneously to form the CHD
Type 2 (Triple confluence) RASD, RPSD and LHD join simultaneously to form the CHD
Type 4 RPSD drains into the common hepatic duct (CHD)
Type 2 (Triple confluence) RASD, RPSD and LHD join simultaneously to form the CHD
Type 2 (Triple confluence) RASD, RPSD and LHD join simultaneously to form the CHD
Type 2 (Triple confluence) RASD, RPSD and LHD join simultaneously to form the CHD
Type 2 (Triple confluence) RASD, RPSD and LHD join simultaneously to form the CHD
Type 4 RPSD drains into the common hepatic duct (CHD)
Type 2 (Triple confluence) RASD, RPSD and LHD join simultaneously to form the CHD
Type 1 RASD joins the RPSD to form the RHD, RHD joins LHD to form the CHD
Type 1 RASD joins the RPSD to form the RHD, RHD joins LHD to form the CHD
Type 1 RASD joins the RPSD to form the RHD, RHD joins LHD to form the CHD
Type 1 RASD joins the RPSD to form the RHD, RHD joins LHD to form the CHD
Type 1 RASD joins the RPSD to form the RHD, RHD joins LHD to form the CHD
Type 1 RASD joins the RPSD to form the RHD, RHD joins LHD to form the CHD

CBD diameter (midsection)	Abbrevations and their meanings
6.2mm	[CBD stands for common bile duct] [CHD stands for common hepatic duct] [RASD stands for Right anterior sectoral duct] [RPSD stands for Right posterior sectoral duct] [RHD stands for right hepatic duct] [LHD stands for Left hepatic ducts] [M stands for Male] [F stands for Female]
4mm	
5mm	
4.8mm	
5.6mm	
4.3mm	
5.1mm	
6.1mm	
6mm	
3.5mm	
4.7mm	
5.5mm	
1.6mm	
5.5mm	
4.5mm	
6mm	
5mm	
2.1mm	
2.6mm	
2mm	
5mm	
2.6mm	
5.3mm	
3.2mm	
4.4mm	
5mm	
1.8mm	
4.5mm	
5mm	
4mm	
6.2mm	

2018 DATA SET

Patient ID	Age	Gall bladder variation (shape, position)	Extrahepatic bile duct variation (cycstic duct)	Intrahepatic bile duct variation	CBD diameter (midsection)	Abbrevations and their meanings
128	81/F	Pear shaped, Normal position	Right lateral union of cystic duct to CHD midway between porta hepatis & ampulla of	Type 3 RPSD joins the LHD ,RASD joins the LHD to form CHD,	6.2mm	[CBD stands for common bile duct] [CHD stands for common hepatic duct] [RASD stands for Right anterior sectoral duct] [RPSD stands for Right posterior sectoral duct] [RHD stands for right hepatic duct] [LHD stands for Left hepatic ducts] [M stands for Male]
129	55/F	Cylindrical, Normal position	High entry	Type 1 RASD joins the RPSD to form the RHD, RHD joins LHD to form the CHD	4mm	
130	44/F	Cylindrical, Normal position	High entry	Type 1 RASD joins the RPSD to form the RHD, RHD joins LHD to form the CHD	5mm	
132	59/M	Cylindrical, Normal position	High entry	Type 1 RASD joins the RPSD to form the RHD, RHD joins LHD to form the CHD	4.8mm	
134	78/F	Cylindrical, Normal position	Low entry	Type 1 RASD joins the RPSD to form the RHD, RHD joins LHD to form the CHD	5.6mm	
137	7/M	Cylindrical, Normal position	Low entry	Type 1 RASD joins the RPSD to form the RHD, RHD joins LHD to form the CHD	4.3mm	
139	52/F	Cylindrical, Normal position	Medial entry (CD crosses posterior to CHD and joins it medially)	Type 2 (Triple confluence) RASD, RPSD and LHD join simultaneously to form the CHD	5.1mm	
140	84/F	Cylindrical, Normal position	Low entry	Type 1 RASD joins the RPSD to form the RHD, RHD joins LHD to form the CHD	6.1mm	
141	24/F	Pear shaped, Normal position	Low entry	Type 4 RPSD drains into the common hepatic duct (CHD)	6mm	
142	46/F	Pear shaped, Normal position	Low entry	Type 1 RASD joins the RPSD to form the RHD, RHD joins LHD to form the CHD	3.5mm	
143	60/F	Pear shaped, Normal position	Low entry	Type 1 RASD joins the RPSD to form the RHD, RHD joins LHD to form the CHD	4.7mm	
144	39/M	Phyrigian cap gallbladder, Normal position	High entry	Type 2 (Triple confluence) RASD, RPSD and LHD join simultaneously to form the CHD	5.5mm	
145	6/M	Cylindrical, Normal position	Right lateral union of cystic duct to CHD midway between porta hepatis & ampulla of	Type 1 RASD joins the RPSD to form the RHD, RHD joins LHD to form the CHD	1.6mm	
148	39/F	Cylindrical, Normal position	Right lateral union of cystic duct to CHD midway between porta hepatis & ampulla of	Type 3 RPSD joins the LHD ,RASD joins the LHD to form CHD,	5.5mm	
150	45/M	Cylindrical, Normal position	Right lateral union of cystic duct to CHD midway between porta hepatis & ampulla of	Type 3 RPSD joins the LHD ,RASD joins the LHD to form CHD,	4.5mm	
154	36/M	Cylindrical, Normal position	Right lateral union of cystic duct to CHD midway between porta hepatis & ampulla of	Type 3 RPSD joins the LHD ,RASD joins the LHD to form CHD,	6mm	
155	52/F	Pear shaped, Normal position	High entry	Type 2 (Triple confluence) RASD, RPSD and LHD join simultaneously to form the CHD	5mm	
156	36/M	Pear shaped, Normal position	High entry	Type 2 (Triple confluence) RASD, RPSD and LHD join simultaneously to form the CHD	2.1mm	
159	48/M	Pear shaped, Normal position	High entry	Type 4 RPSD drains into the common hepatic duct (CHD)	2.6mm	
160	17/F	Pear shaped, Normal position	High entry	Type 2 (Triple confluence) RASD, RPSD and LHD join simultaneously to form the CHD	2mm	
161	34/F	Pear shaped, Normal position	Low entry	Type 2 (Triple confluence) RASD, RPSD and LHD join simultaneously to form the CHD	5mm	
162	3/M	Cylindrical, Normal position	Low entry	Type 2 (Triple confluence) RASD, RPSD and LHD join simultaneously to form the CHD	2.6mm	
163	63/F	Cylindrical, Normal position	Low entry	Type 2 (Triple confluence) RASD, RPSD and LHD join simultaneously to form the CHD	5.3mm	
164	42/M	Cylindrical, Normal position	Right lateral union of cystic duct to CHD midway between porta hepatis & ampulla of	Type 4 RPSD drains into the common hepatic duct (CHD)	3.2mm	
165	52/M	Cylindrical, Normal position	Right lateral union of cystic duct to CHD midway between porta hepatis & ampulla of	Type 2 (Triple confluence) RASD, RPSD and LHD join simultaneously to form the CHD	4.4mm	
166	46/F	Pear shaped, Normal position	Right lateral union of cystic duct to CHD midway between porta hepatis & ampulla of	Type 1 RASD joins the RPSD to form the RHD, RHD joins LHD to form the CHD	5mm	
167	42/M	Pear shaped, Normal position	Right lateral union of cystic duct to CHD midway between porta hepatis & ampulla of	Type 1 RASD joins the RPSD to form the RHD, RHD joins LHD to form the CHD	1.8mm	
168	69/F	Pear shaped, Normal position	Right lateral union of cystic duct to CHD midway between porta hepatis & ampulla of	Type 1 RASD joins the RPSD to form the RHD, RHD joins LHD to form the CHD	4.5mm	
169	38/F	Pear shaped, Normal position	Right lateral union of cystic duct to CHD midway between porta hepatis & ampulla of	Type 1 RASD joins the RPSD to form the RHD, RHD joins LHD to form the CHD	5mm	
170	55/F	Pear shaped, Normal position	High entry	Type 1 RASD joins the RPSD to form the RHD, RHD joins LHD to form the CHD	4mm	
171	81/F	Pear shaped, Normal position	High entry	Type 1 RASD joins the RPSD to form the RHD, RHD joins LHD to form the CHD	6.2mm	
