# Supplementary material for: Anatomical Variations of the Gallbladder and Bile Ducts: An MRI Study
Source: Int J Hepatol. 2024 Oct 19;2024:3877814. doi: 10.1155/2024/3877814 (PMC11512644; doi:10.1155/2024/3877814)
Supplement: Supporting Information 3 — 2019 data set PDF file which contains data collected from MRCP images and reports of patients who visited Kampala MRI Centre in the year 2019. [file 3877814.f3.pdf]

2019 DATA SET

Patient I	Age	Gall bladder variation (shape, position)
174	65/M	Cylindrical, Normal position
175	65/F	Cylindrical, Normal position
176	49F	Cylindrical, Normal position
178	35/F	Cylindrical, Normal position
179	74/M	Phyrigian cap gallbladder, Normal position
182	47/F	Pear shaped, Normal position
185	11/M	Pear shaped, Normal position
186	39/F	Phyrigian cap gallbladder, Normal position
187	48/F	Pear shaped, Normal position
188	39/M	Cylindrical, Normal position
189	32/F	Cylindrical, Normal position
190	59/M	Cylindrical, Normal position
194	55/M	Phyrigian cap gallbladder, Normal position
199	35/F	Pear shaped, Normal position
200	70/F	Pear shaped, Normal position
201	35/F	Pear shaped, Normal position

Extrahepatic bile duct variation
Right lateral union of cystic duct to CHD midway between porta hepatis & ampulla of vatter
Right lateral union of cystic duct to CHD midway between porta hepatis & ampulla of vatter
Right lateral union of cystic duct to CHD midway between porta hepatis & ampulla of vatter
Right lateral union of cystic duct to CHD midway between porta hepatis & ampulla of vatter
Right lateral union of cystic duct to CHD midway between porta hepatis & ampulla of vatter
Right lateral union of cystic duct to CHD midway between porta hepatis & ampulla of vatter
Medial entry (CD crosses posterior to CHD and joins it medially)
Low entry
Low entry
Low entry
Right lateral union of cystic duct to CHD midway between porta hepatis & ampulla of vatter
Right lateral union of cystic duct to CHD midway between porta hepatis & ampulla of vatter
Right lateral union of cystic duct to CHD midway between porta hepatis & ampulla of vatter
High entry
High entry
High entry

Intrahepatic bile duct variation
Type 1 RASD joins the RPSD to form the RHD, RHD joins LHD to form the CHD
Type 1 RASD joins the RPSD to form the RHD, RHD joins LHD to form the CHD
Type 1 RASD joins the RPSD to form the RHD, RHD joins LHD to form the CHD
Type 1 RASD joins the RPSD to form the RHD, RHD joins LHD to form the CHD
Type 2 (Triple confluence) RASD, RPSD and LHD join simultaneously to form the CHD
Type 4 RPSD drains into the common hepatic duct (CHD)
Type 3 RPSD joins the LHD ,RASD joins the LHD to form CHD,
Type 3 RPSD joins the LHD ,RASD joins the LHD to form CHD,
Type 3 RPSD joins the LHD ,RASD joins the LHD to form CHD,
Type 2 (Triple confluence) RASD, RPSD and LHD join simultaneously to form the CHD
Type 2 (Triple confluence) RASD, RPSD and LHD join simultaneously to form the CHD
Type 4 RPSD drains into the common hepatic duct (CHD)
Type 2 (Triple confluence) RASD, RPSD and LHD join simultaneously to form the CHD
Type 2 (Triple confluence) RASD, RPSD and LHD join simultaneously to form the CHD
Type 2 (Triple confluence) RASD, RPSD and LHD join simultaneously to form the CHD
Type 2 (Triple confluence) RASD, RPSD and LHD join simultaneously to form the CHD

CBD diameter (midsection)
3.4mm
5mm
6mm
3.8mm
3mm
6.7mm
3mm
3.9mm
2.3mm
4.8mm
5.6mm
4mm
5.6mm
6mm
4mm
5.6mm

2019 DATA SET

Patient	Age	Gall bladder variation (shape, position)	Extrahepatic bile duct variation	Intrahepatic bile duct variation	CBD diameter (midsection)
174	65/M	Cylindrical, Normal position	Right lateral union of cystic duct to CHD midway between porta hepatis & ampulla of vatter	Type 1 RASD joins the RPSD to form the RHD, RHD joins LHD to form the CHD	3.4mm
175	65/F	Cylindrical, Normal position	Right lateral union of cystic duct to CHD midway between porta hepatis & ampulla of vatter	Type 1 RASD joins the RPSD to form the RHD, RHD joins LHD to form the CHD	5mm
176	49F	Cylindrical, Normal position	Right lateral union of cystic duct to CHD midway between porta hepatis & ampulla of vatter	Type 1 RASD joins the RPSD to form the RHD, RHD joins LHD to form the CHD	6mm
178	35/F	Cylindrical, Normal position	Right lateral union of cystic duct to CHD midway between porta hepatis & ampulla of vatter	Type 1 RASD joins the RPSD to form the RHD, RHD joins LHD to form the CHD	3.8mm
179	74/M	Phyrigian cap gallbladder, Normal position	Right lateral union of cystic duct to CHD midway between porta hepatis & ampulla of vatter	Type 2 (Triple confluence) RASD, RPSD and LHD join simultaneously to form the Cl	3mm
182	47/F	Pear shaped, Normal position	Right lateral union of cystic duct to CHD midway between porta hepatis & ampulla of vatter	Type 4 RPSD drains into the common hepatic duct (CHD)	6.7mm
185	11/M	Pear shaped, Normal position	Medial entry (CD crosses posterior to CHD and joins it medially)	Type 3 RPSD joins the LHD ,RASD joins the LHD to form CHD,	3mm
186	39/F	Phyrigian cap gallbladder, Normal position	Low entry	Type 3 RPSD joins the LHD ,RASD joins the LHD to form CHD,	3.9mm
187	48/F	Pear shaped, Normal position	Low entry	Type 3 RPSD joins the LHD ,RASD joins the LHD to form CHD,	2.3mm
188	39/M	Cylindrical, Normal position	Low entry	Type 2 (Triple confluence) RASD, RPSD and LHD join simultaneously to form the Cl	4.8mm
189	32/F	Cylindrical, Normal position	Right lateral union of cystic duct to CHD midway between porta hepatis & ampulla of vatter	Type 2 (Triple confluence) RASD, RPSD and LHD join simultaneously to form the Cl	5.6mm
190	59/M	Cylindrical, Normal position	Right lateral union of cystic duct to CHD midway between porta hepatis & ampulla of vatter	Type 4 RPSD drains into the common hepatic duct (CHD)	4mm
194	55/M	Phyrigian cap gallbladder, Normal position	Right lateral union of cystic duct to CHD midway between porta hepatis & ampulla of vatter	Type 2 (Triple confluence) RASD, RPSD and LHD join simultaneously to form the Cl	5.6mm
199	35/F	Pear shaped, Normal position	High entry	Type 2 (Triple confluence) RASD, RPSD and LHD join simultaneously to form the Cl	6mm
200	70/F	Pear shaped, Normal position	High entry	Type 2 (Triple confluence) RASD, RPSD and LHD join simultaneously to form the Cl	4mm
201	35/F	Pear shaped, Normal position	High entry	Type 2 (Triple confluence) RASD, RPSD and LHD join simultaneously to form the Cl	5.6mm
