# Supplementary material for: Anatomical Variations of the Gallbladder and Bile Ducts: An MRI Study
Source: Int J Hepatol. 2024 Oct 19;2024:3877814. doi: 10.1155/2024/3877814 (PMC11512644; doi:10.1155/2024/3877814)
Supplement: Supporting Information 4 — 2020 data set PDF file which contains data collected from MRCP images and reports of patients who visited Kampala MRI Centre in the year 2020. [file 3877814.f4.pdf]

2020 DATA SET		
Patient ID	Age	Gall bladder variation (shape, position)
202	62,F	Pear shaped, Normal position
203	45,F	Cylindrical, Normal position
204	64,F	Cylindrical, Normal position
209	54,M	Cylindrical, Normal position
222	49,F	Cylindrical, Horizontal position
223	08,F	Cylindrical, Horizontal position
235	82,F	Cylindrical, Horizontal position
236	44,M	Cylindrical, Horizontal position
238	60,F	Pear shaped, Normal position
240	06,F	Pear shaped, Normal position
241	73,F	Phyrigian cap gallbladder, Normal position
244	37 ,M	Cylindrical, Normal position
245	35,F	Cylindrical, Normal position
246	17,M	Cylindrical, Normal position
247	57,M	Cylindrical, Normal position
250	44,F	Pear shaped, Normal position
251	68,F	Pear shaped, Normal position
252	80,F	Pear shaped, Normal position
253	50,M	Pear shaped, Normal position
256	42,F	Pear shaped, Normal position
270	59,M	Cylindrical, Normal position
277	36,F	Cylindrical, Normal position
278	40,M	Cylindrical, Normal position
280	66,M	Cylindrical, Normal position
281	66,F	Cylindrical, Normal position
282	36 ,M	Cylindrical, Normal position
283	45,M	Pear shaped, Normal position
284	43,M	Pear shaped, Normal position
285	40,M	Pear shaped, Normal position
286	45,M	Pear shaped, Normal position
287	60,F	Pear shaped, Normal position
289	63,F	Pear shaped, Normal position
290	65,F	Pear shaped, Normal position
300	60,F	Pear shaped, Normal position
301	45,M	Pear shaped, Normal position
302	61,F	Phyrigian cap gallbladder
306	44,M	Phyrigian cap gallbladder
308	60,F	Phyrigian cap gallbladder
309	43,M	Cylindrical, Normal position
310	41,F	Cylindrical, Normal position
312	60,F	Cylindrical, Normal position
313	43,F	Cylindrical, Normal position
314	45,F	Cylindrical, Normal position
315	40,F	Pear shaped, Normal position

**Extrahepatic bile duct variation (cycstic duct)**
Right lateral union of cystic duct to CHD midway between porta hepatis & ampulla of vatter
Right lateral union of cystic duct to CHD midway between porta hepatis & ampulla of vatter
Right lateral union of cystic duct to CHD midway between porta hepatis & ampulla of vatter
Right lateral union of cystic duct to CHD midway between porta hepatis & ampulla of vatter
Low entry
Low entry
Medial entry (CD crosses posterior to CHD and joins it medially)
Low entry
Low entry
Low entry
High entry
Right lateral union of cystic duct to CHD midway between porta hepatis & ampulla of vatter
Right lateral union of cystic duct to CHD midway between porta hepatis & ampulla of vatter
Right lateral union of cystic duct to CHD midway between porta hepatis & ampulla of vatter
Right lateral union of cystic duct to CHD midway between porta hepatis & ampulla of vatter
High entry
High entry
High entry
High entry
Low entry
Low entry
Low entry
Right lateral union of cystic duct to CHD midway between porta hepatis & ampulla of vatter
Right lateral union of cystic duct to CHD midway between porta hepatis & ampulla of vatter
Right lateral union of cystic duct to CHD midway between porta hepatis & ampulla of vatter
Right lateral union of cystic duct to CHD midway between porta hepatis & ampulla of vatter
Right lateral union of cystic duct to CHD midway between porta hepatis & ampulla of vatter
Right lateral union of cystic duct to CHD midway between porta hepatis & ampulla of vatter
Right lateral union of cystic duct to CHD midway between porta hepatis & ampulla of vatter
Right lateral union of cystic duct to CHD midway between porta hepatis & ampulla of vatter
Right lateral union of cystic duct to CHD midway between porta hepatis & ampulla of vatter
Right lateral union of cystic duct to CHD midway between porta hepatis & ampulla of vatter
High entry
High entry
High entry
Medial entry (CD crosses posterior to CHD and joins it medially)
Low entry
High entry
Low entry
Right lateral union of cystic duct to CHD midway between porta hepatis & ampulla of vatter
Right lateral union of cystic duct to CHD midway between porta hepatis & ampulla of vatter
Right lateral union of cystic duct to CHD midway between porta hepatis & ampulla of vatter
Right lateral union of cystic duct to CHD midway between porta hepatis & ampulla of vatter
Low entry

**Intrahepatic bile duct variation**
Type 3 RPSD joins the LHD ,RASD joins the LHD to form CHD,
Type 1 RASD joins the RPSD to form the RHD, RHD joins LHD to form the CHD
Type 1 RASD joins the RPSD to form the RHD, RHD joins LHD to form the CHD
Type 1 RASD joins the RPSD to form the RHD, RHD joins LHD to form the CHD
Type 1 RASD joins the RPSD to form the RHD, RHD joins LHD to form the CHD
Type 1 RASD joins the RPSD to form the RHD, RHD joins LHD to form the CHD
Type 2 (Triple confluence) RASD, RPSD and LHD join simultaneously to form the CHD
Type 1
Type 1
Type 1
Type 2 (Triple confluence) RASD, RPSD and LHD join simultaneously to form the CHD
Type 1
Type 3 RPSD joins the LHD ,RASD joins the LHD to form CHD,
Type 3 RPSD joins the LHD ,RASD joins the LHD to form CHD,
Type 3 RPSD joins the LHD ,RASD joins the LHD to form CHD,
Type 2 (Triple confluence) RASD, RPSD and LHD join simultaneously to form the CHD
Type 2 (Triple confluence) RASD, RPSD and LHD join simultaneously to form the CHD
Type 2 (Triple confluence) RASD, RPSD and LHD join simultaneously to form the CHD
Type 2 (Triple confluence) RASD, RPSD and LHD join simultaneously to form the CHD
Type 2 (Triple confluence) RASD, RPSD and LHD join simultaneously to form the CHD
Type 2 (Triple confluence) RASD, RPSD and LHD join simultaneously to form the CHD
Type 2 (Triple confluence) RASD, RPSD and LHD join simultaneously to form the CHD
Type 2 (Triple confluence) RASD, RPSD and LHD join simultaneously to form the CHD
Type 2 (Triple confluence) RASD, RPSD and LHD join simultaneously to form the CHD
Type 1 RASD joins the RPSD to form the RHD, RHD joins LHD to form the CHD
Type 1 RASD joins the RPSD to form the RHD, RHD joins LHD to form the CHD
Type 1 RASD joins the RPSD to form the RHD, RHD joins LHD to form the CHD
Type 1 RASD joins the RPSD to form the RHD, RHD joins LHD to form the CHD
Type 1 RASD joins the RPSD to form the RHD, RHD joins LHD to form the CHD
Type 1 RASD joins the RPSD to form the RHD, RHD joins LHD to form the CHD
Type 1 RASD joins the RPSD to form the RHD, RHD joins LHD to form the CHD
Type 1 RASD joins the RPSD to form the RHD, RHD joins LHD to form the CHD
Type 1 RASD joins the RPSD to form the RHD, RHD joins LHD to form the CHD
Type 1 RASD joins the RPSD to form the RHD, RHD joins LHD to form the CHD
Type 2 (Triple confluence) RASD, RPSD and LHD join simultaneously to form the CHD
Type 2 (Triple confluence) RASD, RPSD and LHD join simultaneously to form the CHD
Type 2 (Triple confluence) RASD, RPSD and LHD join simultaneously to form the CHD
Type 2 (Triple confluence) RASD, RPSD and LHD join simultaneously to form the CHD
type 4
type 3 RPSD joins the LHD ,RASD joins the LHD to form CHD,
type 3 RPSD joins the LHD ,RASD joins the LHD to form CHD,
type 3 RPSD joins the LHD ,RASD joins the LHD to form CHD,
type 3 RPSD joins the LHD ,RASD joins the LHD to form CHD,
Type 1 RASD joins the RPSD to form the RHD, RHD joins LHD to form the CHD

**CBD diameter (midsection)**	
3.9mm	
4mm	
4.1mm	
5.8mm	
2.5mm	
3mm	
5.7mm	
3mm	
4mm	
3mm	
5mm	
3.3mm	
3.7mm	
3mm	
6mm	
6mm	
3.8mm	
3mm	
3mm	
5mm	
5.5mm	
4.3mm	
2.5mm	
5.7mm	
4.4mm	
3.1mm	
4.6mm	
4.3mm	
4.4mm	
4.6mm	
5mm	
5.3mm	
5.6mm	
5.8mm	
4mm	
5.5mm	
3.9mm	
5.7mm	
4.5mm	
5mm	
5.6mm	
4.4mm	
5mm	
4.5mm	

1	**2020 DATA SET**					
2	**Patient ID**	**Age**	**Gall bladder variation (shape, posit**	**Extrahepatic bile duct variation (cycstic duct)**	**Intrahepatic bile duct variation**	**CBD diameter (midsection)**
3	202	62,F	Pear shaped, Normal position	Right lateral union of cystic duct to CHD midway between porta hepatis & ampulla of vatter	Type 3 RPSD joins the LHD ,RASD joins the LHD to form CHD,	3.9mm
4	203	45,F	Cylindrical, Normal position	Right lateral union of cystic duct to CHD midway between porta hepatis & ampulla of vatter	Type 1RASD joins the RPSD to form the RHD, RHD joins LHD to form the CHD	4mm
5	204	64,F	Cylindrical, Normal position	Right lateral union of cystic duct to CHD midway between porta hepatis & ampulla of vatter	Type 1RASD joins the RPSD to form the RHD, RHD joins LHD to form the CHD	4.1mm
6	209	54,M	Cylindrical, Normal position	Right lateral union of cystic duct to CHD midway between porta hepatis & ampulla of vatter	Type 1RASD joins the RPSD to form the RHD, RHD joins LHD to form the CHD	5.8mm
7	222	49,F	Cylindrical, Horizontal position	Low entry	Type 1RASD joins the RPSD to form the RHD, RHD joins LHD to form the CHD	2.5mm
8	223	08,F	Cylindrical, Horizontal position	Low entry	Type 1RASD joins the RPSD to form the RHD, RHD joins LHD to form the CHD	3mm
9	235	82,F	Cylindrical, Horizontal position	Medial entry (CD crosses posterior to CHD and joins it medially)	Type 2 (Triple confluence) RASD, RPSD and LHD join simultaneously to form the CHD	5.7mm
10	236	44,M	Cylindrical, Horizontal position	Low entry	Type 1	3mm
11	238	60,F	Pear shaped, Normal position	Low entry	Type 1	4mm
12	240	06,F	Pear shaped, Normal position	Low entry	Type 1	3mm
13	241	73,F	Phyrigian cap gallbladder, Normal position	High entry	Type 2 (Triple confluence) RASD, RPSD and LHD join simultaneously to form the CHD	5mm
14	244	37 ,M	Cylindrical, Normal position	Right lateral union of cystic duct to CHD midway between porta hepatis & ampulla of vatter	Type 1	3.3mm
15	245	35,F	Cylindrical, Normal position	Right lateral union of cystic duct to CHD midway between porta hepatis & ampulla of vatter	Type 3 RPSD joins the LHD ,RASD joins the LHD to form CHD,	3.7mm
16	246	17,M	Cylindrical, Normal position	Right lateral union of cystic duct to CHD midway between porta hepatis & ampulla of vatter	Type 3 RPSD joins the LHD ,RASD joins the LHD to form CHD,	3mm
17	247	57,M	Cylindrical, Normal position	Right lateral union of cystic duct to CHD midway between porta hepatis & ampulla of vatter	Type 3 RPSD joins the LHD ,RASD joins the LHD to form CHD,	6mm
18	250	44,F	Pear shaped, Normal position	High entry	Type 2 (Triple confluence) RASD, RPSD and LHD join simultaneously to form the CHD	6mm
19	251	68,F	Pear shaped, Normal position	High entry	Type 2 (Triple confluence) RASD, RPSD and LHD join simultaneously to form the CHD	3.8mm
20	252	80,F	Pear shaped, Normal position	High entry	Type 2 (Triple confluence) RASD, RPSD and LHD join simultaneously to form the CHD	3mm
21	253	50,M	Pear shaped, Normal position	High entry	Type 2 (Triple confluence) RASD, RPSD and LHD join simultaneously to form the CHD	3mm
22	256	42,F	Pear shaped, Normal position	Low entry	Type 2 (Triple confluence) RASD, RPSD and LHD join simultaneously to form the CHD	5mm
23	270	59,M	Cylindrical, Normal position	Low entry	Type 2 (Triple confluence) RASD, RPSD and LHD join simultaneously to form the CHD	5.5mm
24	277	36,F	Cylindrical, Normal position	Low entry	Type 2 (Triple confluence) RASD, RPSD and LHD join simultaneously to form the CHD	4.3mm
25	278	40,M	Cylindrical, Normal position	Right lateral union of cystic duct to CHD midway between porta hepatis & ampulla of vatter	Type 2 (Triple confluence) RASD, RPSD and LHD join simultaneously to form the CHD	2.5mm
26	280	66,M	Cylindrical, Normal position	Right lateral union of cystic duct to CHD midway between porta hepatis & ampulla of vatter	Type 2 (Triple confluence) RASD, RPSD and LHD join simultaneously to form the CHD	5.7mm
27	281	66,F	Cylindrical, Normal position	Right lateral union of cystic duct to CHD midway between porta hepatis & ampulla of vatter	Type 1RASD joins the RPSD to form the RHD, RHD joins LHD to form the CHD	4.4mm
28	282	36 ,M	Cylindrical, Normal position	Right lateral union of cystic duct to CHD midway between porta hepatis & ampulla of vatter	Type 1RASD joins the RPSD to form the RHD, RHD joins LHD to form the CHD	3.1mm
29	283	45,M	Pear shaped, Normal position	Right lateral union of cystic duct to CHD midway between porta hepatis & ampulla of vatter	Type 1RASD joins the RPSD to form the RHD, RHD joins LHD to form the CHD	4.6mm
30	284	43,M	Pear shaped, Normal position	Right lateral union of cystic duct to CHD midway between porta hepatis & ampulla of vatter	Type 1RASD joins the RPSD to form the RHD, RHD joins LHD to form the CHD	4.3mm
