# Supplementary material for: Anatomical Variations of the Gallbladder and Bile Ducts: An MRI Study
Source: Int J Hepatol. 2024 Oct 19;2024:3877814. doi: 10.1155/2024/3877814 (PMC11512644; doi:10.1155/2024/3877814)
Supplement: Supporting Information 5 — 2021 data set PDF file which contains data collected from MRCP images and reports of patients who visited Kampala MRI Centre in the year 2021. [file 3877814.f5.pdf]

	2021 DATA SET	
Patient ID	Age	Gall bladder variation (shape, position)
316	52,F	Cylindrical, Normal position
317	38,M	Pear shaped, Normal position
320	66,F	Phyrigian cap gallbladder
321	46,F	Phyrigian cap gallbladder
324	62,M	Phyrigian cap gallbladder
326	56,F	Cylindrical, Normal position
328	54,M	Phyrigian cap gallbladder
32	35,F	Pear shaped, Normal position
330	44,M	Pear shaped, Normal position
331	40,F	cylindrical, Normal position
332	51,F	cylindrical, Normal position
333	76,F	cylindrical, Normal position
334	56,M	Pear shaped, Normal position
335	56,F	Pear shaped, Normal position
336	43,M	Phyrigian cap gallbladder
337	48,M	Phyrigian cap gallbladder
338	30,M	Phyrigian cap gallbladder
339	60,F	Phyrigian cap gallbladder
340	20,M	Pear shaped, Normal position
342	63,F	Pear shaped, Normal position
345	40,M	Pear shaped, Normal position
346	33,F	Pear shaped, Normal position
348	68,M	Pear shaped, Normal position
349	42,F	Pear shaped, Normal position
350	63,M	Cylindrical, Normal position
351	41,F	Cylindrical, Horizontal position
352	28,M	Pear shaped, Normal position
353	32,F	Pear shaped, Normal position
354	28,M	Pear shaped, Normal position
355	82,F	Pear shaped, Normal position
356	56,M	Pear shaped, normal position
357	53,F	Pear shaped, normal position
358	35,M	Pear shaped, normal position
359	47,F	Pear shaped, normal position
360	38,M	Pear shaped, Normal position
361	72,F	Pear shaped, normal position
362	62,F	Pear shaped, Normal position
363	67,M	Pear shaped, normal position
364	56,F	Pear shaped, normal position
365	65/F	Pear shaped, Normal position
366	49F	Pear shaped, Normal position
367	35/F	Pear shaped, Normal position
368	74/M	Pear shaped, Normal position
369	47/F	Pear shaped, normal position

370	11/M	Pear shaped, normal position
371	39/F	Pear shaped, normal position
372	48/F	Pear shaped, normal position
373	39/M	Pear shaped, Normal position
374	32/F	Pear shaped, normal position

**Extrahepatic bile duct variation**
Low entry
Low entry
Right lateral union of cystic duct to CHD midway between porta hepatis & ampulla of vatter
Right lateral union of cystic duct to CHD midway between porta hepatis & ampulla of vatter
Right lateral union of cystic duct to CHD midway between porta hepatis & ampulla of vatter
High entry
High entry
High entry
Right lateral union of cystic duct to CHD midway between porta hepatis & ampulla of vatter
Right lateral union of cystic duct to CHD midway between porta hepatis & ampulla of vatter
Right lateral union of cystic duct to CHD midway between porta hepatis & ampulla of vatter
Right lateral union of cystic duct to CHD midway between porta hepatis & ampulla of vatter
Right lateral union of cystic duct to CHD midway between porta hepatis & ampulla of vatter
Right lateral union of cystic duct to CHD midway between porta hepatis & ampulla of vatter
Right lateral union of cystic duct to CHD midway between porta hepatis & ampulla of vatter
Medial entry
Low entry
Right lateral union of cystic duct to CHD midway between porta hepatis & ampulla of vatter
Right lateral union of cystic duct to CHD midway between porta hepatis & ampulla of vatter
Right lateral union of cystic duct to CHD midway between porta hepatis & ampulla of vatter
Right lateral union of cystic duct to CHD midway between porta hepatis & ampulla of vatter
Right lateral union of cystic duct to CHD midway between porta hepatis & ampulla of vatter
Right lateral union of cystic duct to CHD midway between porta hepatis & ampulla of vatter
Right lateral union of cystic duct to CHD midway between porta hepatis & ampulla of vatter
Right lateral union of cystic duct to CHD midway between porta hepatis & ampulla of vatter
Medial entry
High entry
Medial entry
Low entry
Low entry
Low entry
High entry
Right lateral union of cystic duct to CHD midway between porta hepatis & ampulla of vatter
Right lateral union of cystic duct to CHD midway between porta hepatis & ampulla of vatter
Right lateral union of cystic duct to CHD midway between porta hepatis & ampulla of vatter
Right lateral union of cystic duct to CHD midway between porta hepatis & ampulla of vatter
Right lateral union of cystic duct to CHD midway between porta hepatis & ampulla of vatter
High entry
Right lateral union of cystic duct to CHD midway between porta hepatis & ampulla of vatter
Right lateral union of cystic duct to CHD midway between porta hepatis & ampulla of vatter
Right lateral union of cystic duct to CHD midway between porta hepatis & ampulla of vatter
Low entry
Right lateral union of cystic duct to CHD midway between porta hepatis & ampulla of vatter
Right lateral union of cystic duct to CHD midway between porta hepatis & ampulla of vatter

High entry
Low entry
Medial entry
High entry
High entry

**Intrahepatic bile duct variation**
Type 1 RASD joins the RPSD to form the RHD, RHD joins LHD to form the CHD
Type 1
Type 1
Type 1
Type 2 (Triple confluence) RASD, RPSD and LHD join simultaneously to form the CHD
Type 1
Type 3 RPSD joins the LHD ,RASD joins the LHD to form CHD,
Type 3 RPSD joins the LHD ,RASD joins the LHD to form CHD,
Type 3 RPSD joins the LHD ,RASD joins the LHD to form CHD,
Type 2 (Triple confluence) RASD, RPSD and LHD join simultaneously to form the CHD
Type 2 (Triple confluence) RASD, RPSD and LHD join simultaneously to form the CHD
Type 2 (Triple confluence) RASD, RPSD and LHD join simultaneously to form the CHD
Type 2 (Triple confluence) RASD, RPSD and LHD join simultaneously to form the CHD
Type 2 (Triple confluence) RASD, RPSD and LHD join simultaneously to form the CHD
Type 2 (Triple confluence) RASD, RPSD and LHD join simultaneously to form the CHD
Type 2 (Triple confluence) RASD, RPSD and LHD join simultaneously to form the CHD
Type 1 RASD joins the RPSD to form the RHD, RHD joins LHD to form the CHD
Type 1 RASD joins the RPSD to form the RHD, RHD joins LHD to form the CHD
Type 1 RASD joins the RPSD to form the RHD, RHD joins LHD to form the CHD
Type 1 RASD joins the RPSD to form the RHD, RHD joins LHD to form the CHD
Type 1 RASD joins the RPSD to form the RHD, RHD joins LHD to form the CHD
Type 1 RASD joins the RPSD to form the RHD, RHD joins LHD to form the CHD
Type 1 RASD joins the RPSD to form the RHD, RHD joins LHD to form the CHD
Type 1 RASD joins the RPSD to form the RHD, RHD joins LHD to form the CHD
Type 1 RASD joins the RPSD to form the RHD, RHD joins LHD to form the CHD
Type 1 RASD joins the RPSD to form the RHD, RHD joins LHD to form the CHD
Type 1 RASD joins the RPSD to form the RHD, RHD joins LHD to form the CHD
Type 1 RASD joins the RPSD to form the RHD, RHD joins LHD to form the CHD
Type 2 (Triple confluence) RASD, RPSD and LHD join simultaneously to form the CHD
Type 2 (Triple confluence) RASD, RPSD and LHD join simultaneously to form the CHD
Type 2 (Triple confluence) RASD, RPSD and LHD join simultaneously to form the CHD
Type 2 (Triple confluence) RASD, RPSD and LHD join simultaneously to form the CHD
Type 2 (Triple confluence) RASD, RPSD and LHD join simultaneously to form the CHD
Type 2 (Triple confluence) RASD, RPSD and LHD join simultaneously to form the CHD
Type 2 (Triple confluence) RASD, RPSD and LHD join simultaneously to form the CHD
Type 2 (Triple confluence) RASD, RPSD and LHD join simultaneously to form the CHD
Type 3 RPSD joins the LHD ,RASD joins the LHD to form CHD,
Type 3 RPSD joins the LHD ,RASD joins the LHD to form CHD,
Type 3 RPSD joins the LHD ,RASD joins the LHD to form CHD,
Type 1 RASD joins the RPSD to form the RHD, RHD joins LHD to form the CHD
Type 1 RASD joins the RPSD to form the RHD, RHD joins LHD to form the CHD
Type 1 RASD joins the RPSD to form the RHD, RHD joins LHD to form the CHD
Type 1 RASD joins the RPSD to form the RHD, RHD joins LHD to form the CHD
Type 2 (Triple confluence) RASD, RPSD and LHD join simultaneously to form the CHD

Type 2 (Triple confluence) RASD, RPSD and LHD join simultaneously to form the CHD
type 4
Type 1 RASD joins the RPSD to form the RHD, RHD joins LHD to form the CHD
Type 1 RASD joins the RPSD to form the RHD, RHD joins LHD to form the CHD
Type 1 RASD joins the RPSD to form the RHD, RHD joins LHD to form the CHD

**CBD diameter (midsection)**
5.5 MM
4.0 MM
6.8 MM
4.0 MM
2.4 MM
2.1 MM
3.0 MM
2.9 MM
4.8 MM
3.5 MM
5.1 MM
5.0 MM
3.0 MM
5.0MM
4.0MM
4.0MM
3.8MM
4.1MM
4.0MM
5.5MM
4.OMM
6.0MM
3.6MM
4.2MM
9MM
7.5MM
7.0MM
4.0MM
4.3MM
7.7MM
5.0MM
4.9MM
3.0MM
8.0MM
3.6MM
8.3MM
4.5MM
6.0MM
5.0MM
5mm
6mm
3.8mm
3mm
6.7mm

3mm
3.9mm
2.3mm
4.8mm
5.6mm

2021 DATA SET

Patient	Age	Gall bladder variation (shape, position)	Extrahepatic bile duct variation	Intrahepatic bile duct variation	CBD diameter (midsection)
316	52,F	Cylindrical, Normal position	Low entry	Type 1 RASD joins the RPSD to form the RHD, RHD joins LHD to form the CHD	5.5 MM
317	38,M	Pear shaped, Normal position	Low entry	Type 1	4.0 MM
320	66,F	Phyrigian cap gallbladder	Right lateral union of cystic duct to CHD midway between porta hepatis & ampulla of vatter	Type 1	6.8 MM
321	46,F	Phyrigian cap gallbladder	Right lateral union of cystic duct to CHD midway between porta hepatis & ampulla of vatter	Type 1	4.0 MM
324	62,M	Phyrigian cap gallbladder	Right lateral union of cystic duct to CHD midway between porta hepatis & ampulla of vatter	Type 2 (Triple confluence) RASD, RPSD and LHD join simultaneously to form the CH	2.4 MM
326	56,F	Cylindrical, Normal position	High entry	Type 1	2.1MM
328	54,M	Phyrigian cap gallbladder	High entry	Type 3 RPSD joins the LHD ,RASD joins the LHD to form CHD,	3.0 MM
32	35,F	Pear shaped, Normal position	High entry	Type 3 RPSD joins the LHD ,RASD joins the LHD to form CHD,	2.9 MM
330	44,M	Pear shaped, Normal position	Right lateral union of cystic duct to CHD midway between porta hepatis & ampulla of vatter	Type 3 RPSD joins the LHD ,RASD joins the LHD to form CHD,	4.8 MM
331	40,F	cylindrical, Normal position	Right lateral union of cystic duct to CHD midway between porta hepatis & ampulla of vatter	Type 2 (Triple confluence) RASD, RPSD and LHD join simultaneously to form the CH	3.5 MM
332	51,F	cylindrical, Normal position	Right lateral union of cystic duct to CHD midway between porta hepatis & ampulla of vatter	Type 2 (Triple confluence) RASD, RPSD and LHD join simultaneously to form the CH	5.1MM
333	76,F	cylindrical, Normal position	Right lateral union of cystic duct to CHD midway between porta hepatis & ampulla of vatter	Type 2 (Triple confluence) RASD, RPSD and LHD join simultaneously to form the CH	5.0 MM
334	56,M	Pear shaped, Normal position	Right lateral union of cystic duct to CHD midway between porta hepatis & ampulla of vatter	Type 2 (Triple confluence) RASD, RPSD and LHD join simultaneously to form the CH	3.0 MM
335	56,F	Pear shaped, Normal position	Right lateral union of cystic duct to CHD midway between porta hepatis & ampulla of vatter	Type 2 (Triple confluence) RASD, RPSD and LHD join simultaneously to form the CH	5.0MM
336	43,M	Phyrigian cap gallbladder	Right lateral union of cystic duct to CHD midway between porta hepatis & ampulla of vatter	Type 2 (Triple confluence) RASD, RPSD and LHD join simultaneously to form the CH	4.0MM
337	48,M	Phyrigian cap gallbladder	Medial entry	Type 2 (Triple confluence) RASD, RPSD and LHD join simultaneously to form the CH	4.0MM
338	30,M	Phyrigian cap gallbladder	Low entry	Type 1 RASD joins the RPSD to form the RHD, RHD joins LHD to form the CHD	3.8MM
339	60,F	Phyrigian cap gallbladder	Right lateral union of cystic duct to CHD midway between porta hepatis & ampulla of vatter	Type 1 RASD joins the RPSD to form the RHD, RHD joins LHD to form the CHD	4.1MM
340	20,M	Pear shaped, Normal position	Right lateral union of cystic duct to CHD midway between porta hepatis & ampulla of vatter	Type 1 RASD joins the RPSD to form the RHD, RHD joins LHD to form the CHD	4.0MM
342	63,F	Pear shaped, Normal position	Right lateral union of cystic duct to CHD midway between porta hepatis & ampulla of vatter	Type 1 RASD joins the RPSD to form the RHD, RHD joins LHD to form the CHD	5.5MM
345	40,M	Pear shaped, Normal position	Right lateral union of cystic duct to CHD midway between porta hepatis & ampulla of vatter	Type 1 RASD joins the RPSD to form the RHD, RHD joins LHD to form the CHD	4.OMM
346	33,F	Pear shaped, Normal position	Right lateral union of cystic duct to CHD midway between porta hepatis & ampulla of vatter	Type 1 RASD joins the RPSD to form the RHD, RHD joins LHD to form the CHD	6.0MM
348	68,M	Pear shaped, Normal position	Right lateral union of cystic duct to CHD midway between porta hepatis & ampulla of vatter	Type 1 RASD joins the RPSD to form the RHD, RHD joins LHD to form the CHD	3.6MM
349	42,F	Pear shaped, Normal position	Right lateral union of cystic duct to CHD midway between porta hepatis & ampulla of vatter	Type 1 RASD joins the RPSD to form the RHD, RHD joins LHD to form the CHD	4.2MM
350	63,M	Cylindrical, Normal position	Right lateral union of cystic duct to CHD midway between porta hepatis & ampulla of vatter	Type 1 RASD joins the RPSD to form the RHD, RHD joins LHD to form the CHD	9MM
351	41,F	Cylindrical, Horizontal position	Medial entry	Type 1 RASD joins the RPSD to form the RHD, RHD joins LHD to form the CHD	7.5MM
352	28,M	Pear shaped, Normal position	High entry	Type 1 RASD joins the RPSD to form the RHD, RHD joins LHD to form the CHD	7.0MM
353	32,F	Pear shaped, Normal position	Medial entry	Type 1 RASD joins the RPSD to form the RHD, RHD joins LHD to form the CHD	4.0MM
354	28,M	Pear shaped, Normal position	Low entry	Type 2 (Triple confluence) RASD, RPSD and LHD join simultaneously to form the CH	4.3MM
355	82,F	Pear shaped, Normal position	Low entry	Type 2 (Triple confluence) RASD, RPSD and LHD join simultaneously to form the CH	7.7MM
356	56,M	Pear shaped, normal position	Low entry	Type 2 (Triple confluence) RASD, RPSD and LHD join simultaneously to form the CH	5.0MM
357	53,F	Pear shaped, normal position	High entry	Type 2 (Triple confluence) RASD, RPSD and LHD join simultaneously to form the CH	4.9MM
358	35,M	Pear shaped, normal position	Right lateral union of cystic duct to CHD midway between porta hepatis & ampulla of vatter	Type 2 (Triple confluence) RASD, RPSD and LHD join simultaneously to form the CH	3.0MM
359	47,F	Pear shaped, normal position	Right lateral union of cystic duct to CHD midway between porta hepatis & ampulla of vatter	Type 2 (Triple confluence) RASD, RPSD and LHD join simultaneously to form the CH	8.0MM
