# Supplementary material for: Anatomical Variations of the Gallbladder and Bile Ducts: An MRI Study
Source: Int J Hepatol. 2024 Oct 19;2024:3877814. doi: 10.1155/2024/3877814 (PMC11512644; doi:10.1155/2024/3877814)
Supplement: Supporting Information 6 — 2022 data set PDF file which contains data collected from MRCP images and reports of patients who visited Kampala MRI Centre in the year 2022. [file 3877814.f6.pdf]

2022 Data set

Patient ID	Age	Gall bladder variation (shape, position)
375	49/F	Pear shaped, Normal position
380	43/F	Pear shaped, Normal position
381	18/M	Pear shaped, Normal position
382	59/F	Pear shaped, Normal position
385	47/F	Pear shaped, Normal position
388	12/F	Pear shaped, Normal position
389	44/F	Pear shaped, Normal position
390	28/M	Pear shaped, Normal position
391	25/M	Pear shaped, Normal position
392	40/M	Cylindrical, Normal position
393	27/M	Cylindrical, Normal position
394	67/F	Cylindrical, Normal position
395	8/F	Phyrigian cap gallbladder
396	40/F	Pear shaped, Normal position
397	28/M	Cylindrical, Horizontal position
398	42/M	Cylindrical, Horizontal position
399	20/F	Cylindrical, Horizontal position
400	47/F	Pear shaped, Normal position
401	63/F	Cylindrical, Normal position
402	42/M	Cylindrical, Normal position
403	52/M	Cylindrical, Normal position
404	46/F	Pear shaped, Normal position
405	42/M	Pear shaped, Normal position
406	69/F	Pear shaped, Normal position
407	38/F	Pear shaped, Normal position
408	55/F	Pear shaped, Normal position
409	81/F	Pear shaped, Normal position
410	66,M	Cylindrical, Normal position
411	66,F	Cylindrical, Normal position
412	36 ,M	Cylindrical, Normal position
413	45,M	Pear shaped, Normal position
414	43,M	Pear shaped, Normal position
415	40,M	Pear shaped, Normal position
416	45,M	Pear shaped, Normal position
417	60,F	Pear shaped, Normal position
418	63,F	Pear shaped, Normal position
419	65,F	Pear shaped, Normal position
420	60,F	Pear shaped, Normal position

Extrahepatic bile duct variation
Right lateral union of cystic duct to CHD midway between porta hepatis & ampulla of vatter
Right lateral union of cystic duct to CHD midway between porta hepatis & ampulla of vatter
Right lateral union of cystic duct to CHD midway between porta hepatis & ampulla of vatter
Right lateral union of cystic duct to CHD midway between porta hepatis & ampulla of vatter
High entry
High entry
High entry
High entry
Low entry
Low entry
Low entry
Low entry
Right lateral union of cystic duct to CHD midway between porta hepatis & ampulla of vatter
Right lateral union of cystic duct to CHD midway between porta hepatis & ampulla of vatter
Right lateral union of cystic duct to CHD midway between porta hepatis & ampulla of vatter
Right lateral union of cystic duct to CHD midway between porta hepatis & ampulla of vatter
High entry
High entry
Low entry
Right lateral union of cystic duct to CHD midway between porta hepatis & ampulla of vatter
Right lateral union of cystic duct to CHD midway between porta hepatis & ampulla of vatter
Right lateral union of cystic duct to CHD midway between porta hepatis & ampulla of vatter
Right lateral union of cystic duct to CHD midway between porta hepatis & ampulla of vatter
Right lateral union of cystic duct to CHD midway between porta hepatis & ampulla of vatter
Right lateral union of cystic duct to CHD midway between porta hepatis & ampulla of vatter
High entry
High entry
Right lateral union of cystic duct to CHD midway between porta hepatis & ampulla of vatter
Right lateral union of cystic duct to CHD midway between porta hepatis & ampulla of vatter
Right lateral union of cystic duct to CHD midway between porta hepatis & ampulla of vatter
Right lateral union of cystic duct to CHD midway between porta hepatis & ampulla of vatter
Right lateral union of cystic duct to CHD midway between porta hepatis & ampulla of vatter
Right lateral union of cystic duct to CHD midway between porta hepatis & ampulla of vatter
Right lateral union of cystic duct to CHD midway between porta hepatis & ampulla of vatter
Right lateral union of cystic duct to CHD midway between porta hepatis & ampulla of vatter
Right lateral union of cystic duct to CHD midway between porta hepatis & ampulla of vatter
High entry
High entry

Intrahepatic bile duct variation
Type 1 RASD joins the RPSD to form the RHD, RHD joins LHD to form the CHD
Type 1
Type 1
Type 1
Type 1
Type 1
Type 1
Type 1
Type 2 (Triple confluence) RASD, RPSD and LHD join simultaneously to form the CHD
Type 1
Type 3 RPSD joins the LHD ,RASD joins the LHD to form CHD,
Type 3 RPSD joins the LHD ,RASD joins the LHD to form CHD,
Type 2 (Triple confluence) RASD, RPSD and LHD join simultaneously to form the CHD
Type 2 (Triple confluence) RASD, RPSD and LHD join simultaneously to form the CHD
Type 2 (Triple confluence) RASD, RPSD and LHD join simultaneously to form the CHD
Type 1 RASD joins the RPSD to form the RHD, RHD joins LHD to form the CHD
Type 1 RASD joins the RPSD to form the RHD, RHD joins LHD to form the CHD
Type 1 RASD joins the RPSD to form the RHD, RHD joins LHD to form the CHD
Type 2 (Triple confluence) RASD, RPSD and LHD join simultaneously to form the CHD
Type 4 RPSD drains into the common hepatic duct (CHD)
Type 2 (Triple confluence) RASD, RPSD and LHD join simultaneously to form the CHD
Type 1 RASD joins the RPSD to form the RHD, RHD joins LHD to form the CHD
Type 1 RASD joins the RPSD to form the RHD, RHD joins LHD to form the CHD
Type 1 RASD joins the RPSD to form the RHD, RHD joins LHD to form the CHD
Type 1 RASD joins the RPSD to form the RHD, RHD joins LHD to form the CHD
Type 1 RASD joins the RPSD to form the RHD, RHD joins LHD to form the CHD
Type 1 RASD joins the RPSD to form the RHD, RHD joins LHD to form the CHD
Type 2 (Triple confluence) RASD, RPSD and LHD join simultaneously to form the CHD
Type 1 RASD joins the RPSD to form the RHD, RHD joins LHD to form the CHD
Type 1 RASD joins the RPSD to form the RHD, RHD joins LHD to form the CHD
Type 1 RASD joins the RPSD to form the RHD, RHD joins LHD to form the CHD
Type 1 RASD joins the RPSD to form the RHD, RHD joins LHD to form the CHD
Type 1 RASD joins the RPSD to form the RHD, RHD joins LHD to form the CHD
Type 1 RASD joins the RPSD to form the RHD, RHD joins LHD to form the CHD
Type 1 RASD joins the RPSD to form the RHD, RHD joins LHD to form the CHD
Type 1 RASD joins the RPSD to form the RHD, RHD joins LHD to form the CHD
Type 1 RASD joins the RPSD to form the RHD, RHD joins LHD to form the CHD
Type 1 RASD joins the RPSD to form the RHD, RHD joins LHD to form the CHD

CBD diameter (midsection)
4mm
6.8mm
2.7mm
4.5mm
6.2mm
2.4mm
6.0mm
4.0mm
3.0mm
2.7mm
4mm
3.5mm
3.0mm
4.0mm
4.2mm
4.0mm
6.0mm
4.2mm
5.3mm
3.2mm
4.4mm
5mm
1.8mm
4.5mm
5mm
4mm
6.2mm
5.7mm
4.4mm
3.1mm
4.6mm
4.3mm
4.4mm
4.6mm
5mm
5.3mm
5.6mm
5.8mm

	A	B	C	D	E	F
1	**2022 Data set**					
2	**Patient ID**	**Age**	**Gall bladder variation (shape, position)**	**Extrahepatic bile duct variation**	**Intrahepatic bile duct variation**	**CBD diameter (midsection)**
3	375	49/F	Pear shaped, Normal position	Right lateral union of cystic duct to CHD midway between porta hepatis & ampulla of vatter	Type 1 RASD joins the RPSD to form the RHD, RHD joins LHD to form the CHD	4mm
4	380	43/F	Pear shaped, Normal position	Right lateral union of cystic duct to CHD midway between porta hepatis & ampulla of vatter	Type 1	6.8mm
5	381	18/M	Pear shaped, Normal position	Right lateral union of cystic duct to CHD midway between porta hepatis & ampulla of vatter	Type 1	2.7mm
6	382	59/F	Pear shaped, Normal position	Right lateral union of cystic duct to CHD midway between porta hepatis & ampulla of vatter	Type 1	4.5mm
7	385	47/F	Pear shaped, Normal position	High entry	Type 1	6.2mm
8	388	12/F	Pear shaped, Normal position	High entry	Type 1	2.4mm
9	389	44/F	Pear shaped, Normal position	High entry	Type 1	6.0mm
10	390	28/M	Pear shaped, Normal position	High entry	Type 1	4.0mm
11	391	25/M	Pear shaped, Normal position	Low entry	Type 2 (Triple confluence) RASD, RPSD and LHD join simultaneously to form the CH	3.0mm
12	392	40/M	Cylindrical, Normal position	Low entry	Type 1	2.7mm
13	393	27/M	Cylindrical, Normal position	Low entry	Type 3 RPSD joins the LHD ,RASD joins the LHD to form CHD,	4mm
14	394	67/F	Cylindrical, Normal position	Low entry	Type 3 RPSD joins the LHD ,RASD joins the LHD to form CHD,	3.5mm
15	395	8/F	Phyrigian cap gallbladder	Right lateral union of cystic duct to CHD midway between porta hepatis & ampulla of vatter	Type 2 (Triple confluence) RASD, RPSD and LHD join simultaneously to form the CH	3.0mm
16	396	40/F	Pear shaped, Normal position	Right lateral union of cystic duct to CHD midway between porta hepatis & ampulla of vatter	Type 2 (Triple confluence) RASD, RPSD and LHD join simultaneously to form the CH	4.0mm
17	397	28/M	Cylindrical, Horizontal position	Right lateral union of cystic duct to CHD midway between porta hepatis & ampulla of vatter	Type 2 (Triple confluence) RASD, RPSD and LHD join simultaneously to form the CH	4.2mm
18	398	42/M	Cylindrical, Horizontal position	Right lateral union of cystic duct to CHD midway between porta hepatis & ampulla of vatter	Type 1 RASD joins the RPSD to form the RHD, RHD joins LHD to form the CHD	4.0mm
19	399	20/F	Cylindrical, Horizontal position	High entry	Type 1 RASD joins the RPSD to form the RHD, RHD joins LHD to form the CHD	6.0mm
20	400	47/F	Pear shaped, Normal position	High entry	Type 1 RASD joins the RPSD to form the RHD, RHD joins LHD to form the CHD	4.2mm
21	401	63/F	Cylindrical, Normal position	Low entry	Type 2 (Triple confluence) RASD, RPSD and LHD join simultaneously to form the CH	5.3mm
22	402	42/M	Cylindrical, Normal position	Right lateral union of cystic duct to CHD midway between porta hepatis & ampulla of vatter	Type 4 RPSD drains into the common hepatic duct (CHD)	3.2mm
23	403	52/M	Cylindrical, Normal position	Right lateral union of cystic duct to CHD midway between porta hepatis & ampulla of vatter	Type 2 (Triple confluence) RASD, RPSD and LHD join simultaneously to form the CH	4.4mm
24	404	46/F	Pear shaped, Normal position	Right lateral union of cystic duct to CHD midway between porta hepatis & ampulla of vatter	Type 1 RASD joins the RPSD to form the RHD, RHD joins LHD to form the CHD	5mm
25	405	42/M	Pear shaped, Normal position	Right lateral union of cystic duct to CHD midway between porta hepatis & ampulla of vatter	Type 1 RASD joins the RPSD to form the RHD, RHD joins LHD to form the CHD	1.8mm
